# Optimizing solar farm interconnection networks using graph theory and metaheuristic algorithms with economic and reliability analysis

**DOI:** 10.1038/s41598-025-18108-5

**Published:** 2025-09-26

**Authors:** Ali Ghias-Nodoushan, Alireza Sedighi-Anaraki, Mohammad Rasoul Jannesar, Hamid Saeedi-Sourck

**Affiliations:** 1 Electrical Engineering Department, Yazd University, Yazd, Iran; 2 Electrical Engineering Department, Technical and Vocational University, Tehran, Iran; 3 Electrical Engineering Department, Yazd University, Yazd, Iran

**Keywords:** Electrical and electronic engineering, Energy infrastructure

## Abstract

As global energy demand continues to rise and the need to transition from fossil fuels becomes increasingly urgent, integrating solar farms efficiently into power grids presents a significant challenge. This study introduces a novel graph-theoretic framework for designing optimal interconnection networks among distributed solar farms. By utilizing Prim’s algorithm to construct a minimum spanning tree, the proposed method effectively reduces transmission losses and infrastructure costs. The performance of this deterministic approach is benchmarked against Particle Swarm Optimization (PSO), a widely applied metaheuristic technique. To assess network robustness under potential line failures, a new graph-based reliability metric is developed. Case studies involving a cluster of solar farms demonstrate that Prim’s algorithm outperforms PSO in minimizing both power losses and capital investment, while also offering higher topological reliability. Although PSO achieves better load balancing, the graph-based approach proves more effective for loss-sensitive and cost-driven design scenarios. The proposed framework naturally accommodates constraints such as terrain limitations and is scalable to hybrid renewable energy systems. By integrating classical graph theory with practical power system considerations, this work offers a computationally efficient and economically viable solution for the optimal physical integration of large-scale solar energy infrastructure. The proposed methodology also lays a foundation for future integration of AI and machine learning techniques to enable dynamic network optimization under uncertainty.

## Introduction

### Motivation

The global imperative to transition toward sustainable energy systems is driven by increasing energy demand, the depletion of fossil fuel resources, and mounting environmental concerns. Hybrid Renewable Energy Systems (HRES), which combine diverse energy sources such as solar, wind, and biomass with advanced storage technologies, including batteries, supercapacitors, and fuel cells, have emerged as a key solution. These integrated systems enhance grid reliability, decrease operational costs, and contribute to environmental sustainability^1–5^. Recent studies demonstrate that multi-metaheuristic optimization approaches can significantly reduce costs and enhance reliability in autonomous green energy systems^6^. Nevertheless, the effective design and operation of such complex systems pose significant challenges due to the inherent intermittency of renewable sources and the multi-dimensional nature of the optimization process, which must balance economic, environmental, and reliability objectives simultaneously.

### Literature review

The optimization of Hybrid Renewable Energy Systems (HRES) heavily depends on metaheuristic algorithms, which have become essential tools for determining optimal resource combinations and component sizing. Widely used approaches include Genetic Algorithms (GA), Particle Swarm Optimization (PSO), the Firefly Algorithm (FA), the Flower Pollination Algorithm (FPA), and the Artificial Bee Colony (ABC), along with various hybridized versions of these techniques^7–12^. Hybrid algorithms, such as the HIWO/PSO combination^10^, have demonstrated superior performance in reducing power outages and lower Levelized Cost of Energy (LCOE) and Total Net Present Cost (TNPC) compared to conventional methods, suggesting indirect energy efficiency gains.These cost advantages have aligned with empirical observations in hybrid renewable systems^13^. More recent techniques, such as PSO-MWWO, have also shown effectiveness in microgrid energy management and power quality enhancement^14^.

In parallel, stochastic optimization methods (e.g., two-stage scenario-based programming) have demonstrated robust capabilities for managing uncertainty in hybrid renewable-storage systems^15^. Notably, recent work by Yang, Jiang, Guo et al. (2025) has advanced predictive capabilities via a spatiotemporal dynamic graph attention network, which effectively captures cross-cluster convergence effects for ultra-short-term forecasting of photovoltaic (PV) clusters^16^.

Extensive research supports the techno-economic feasibility of a wide array of HRES configurations. For example, solar–biomass systems have demonstrated improved reliability and cost-effectiveness across various geographic regions^2,9,17^. Moreover, optimized microgrids particularly PV-Battery (PV-BTM-BESS) systems have been designed to meet objectives such as energy arbitrage, peak shaving, and maximization of solar self-consumption^18^. In this context, Liu, Lu, Yang et al. (2025) proposed a blockchain-based framework to enhance fairness in energy trading between interconnected microgrids^19^. Economic indicators such as TNPC and LCOE consistently identify solar–biomass combinations as among the most favorable options^9^.

The breadth of this research encompasses both grid-connected^18,20–32^ and off-grid systems^24,26,28,33–37^, along with specialized applications such as electric vehicle (EV) charging infrastructure^38–40^ and fuel cell–based hybrid setups^30,38,41^. These studies often employ advanced, multi-objective optimization techniques, including hybridized algorithms such as Genetic-FPA, Simulated Annealing (SA), NSGA-II, MOPSO, and MOEA/D. Additionally, research into optimal battery technologies for grid-connected systems has focused on minimizing the Cost of Energy (COE), increasing the Internal Rate of Return (IRR), and shortening payback periods^42,43^.

Beyond system configuration, a significant body of work addresses the management of uncertain factors in both energy generation and consumption an inherent challenge in HRES planning and operation. This issue has been tackled using various strategies, adaptive control algorithms for stand-alone systems^44^, stochastic optimization methods for wind-solar microgrids^45^, robust decision-making frameworks like Information Gap Decision Theory (IGDT)^46^, and spatiotemporal load shifting strategies designed to mitigate renewable energy curtailment^47^. AI-based techniques like ANN are emerging for renewable energy management under uncertainty, while classical optimization methods address LCOE minimization in wind farms^48^ and scenario-based PV system design^48–51^. Intelligent control techniques are also being designed for stand-alone hybrid systems operating under high uncertainty^52^. For instance, the spatiotemporal forecasting method by Yang, Jiang, Guo et al. (2025) directly addresses short-term solar intermittency challenges during rapid cloud cover transitions^16^.

Complementing uncertainty mitigation, integrated sustainability assessment frameworks provide deeper insights into long-term impacts. Life cycle assessment (LCA) combined with system-level optimization has revealed pathways for reducing carbon emissions by up to 35%^53^, while novel graph-based methods have been proposed to improve reliability assessment accuracy^54^.Technical optimizations such as component sizing and advanced energy management can significantly enhance sustainability and cost-efficiency in fuel cell vehicles, with hydrogen consumption reductions of ~ 19%^41^.Relatedly, the blockchain model proposed by Liu et al. (2025) facilitates decentralized, policy-compliant energy marketplaces through fairness-constrained smart contracts and optimized trading protocols^19^.

Finally, multi-objective optimization continues to constitute a central theme in HRES research. These studies aim to simultaneously minimize costs, reduce emissions, and enhance system reliability typically measured by metrics such as Loss of Load Probability (LOLP)^11,12,33,34,55–60^. Case studies demonstrate that co-optimization strategies balancing cost and carbon footprint yield more sustainable and economically viable energy solutions. For grid-connected PV/wind/hydro-storage hybrids, this approach enhances resilience while reducing emissions^59^. Similarly, in EV charging systems, multi-objective optimization achieves optimal cost-carbon tradeoffs^60^.

### Research gap

Despite the extensive research on optimizing Hybrid Renewable Energy Systems (HRES), a critical gap remains largely unexplored. Most prior studies concentrate primarily on optimizing the capacity and operational strategies of individual renewable resources or fixed hybrid configurations. While the performance of standalone systems has been well analyzed, the foundational challenge of identifying optimal physical interconnection strategies for building efficient and reliable smart hybrid networks particularly from spatially dispersed resources like solar farms has received limited attention.

Advanced approaches for uncertainty management^45,46,50^ and multi-criteria decision analysis^53^ offer strong capabilities within their respective scopes. However, they do not address the deterministic network design problem central to physically connecting discrete generation nodes operating at nominal capacities. Specifically, these methods lack a systematic mechanism to optimize the trade-off between interconnection infrastructure costs and topological reliability across distributed renewable networks.

### Contribution

To bridge this gap, the present study introduces a novel optimization-based methodology explicitly designed for the physical interconnection of distributed solar farms with predetermined capacities. The proposed framework integrates graph theory with Prim’s algorithm and Particle Swarm Optimization (PSO) to determine the most efficient and reliable network topology. The main objectives are twofold:

1. Minimize the total cost of transmission infrastructure required for interconnecting the solar farms.
2. Maximize the topological reliability of the network using a newly proposed graph-theoretic reliability metric.

All feasible interconnection scenarios are modeled as complete graphs. The performance of Prim’s algorithm is evaluated under varying operational conditions, while PSO is implemented to optimize two distinct objectives: minimizing path loss and minimizing overall path length.

Beyond topological optimization, the proposed framework incorporates comprehensive economic analysis, examining the total investment required for each network design. Furthermore, uncertainty modeling is employed to assess the sensitivity of each topology to fluctuations in operational variables, offering a holistic solution to the HRES network design problem.

### Paper structure

The remainder of this paper is structured as follows:

- Section “Methodology” introduces essential concepts in graph theory, along with the fundamental algorithms used specifically Prim’s algorithm and Particle Swarm Optimization (PSO).
- Section “System under study and prim’s algorithm implementation” models all potential interconnection scenarios among solar farms as complete graphs. It evaluates the performance of Prim’s algorithm under three distinct operational scenarios and applies PSO to optimize objective functions related to power loss and total transmission length.
- Section “Economic estimation and reliability analysis” presents a comparative cost analysis of the various network topologies. It also introduces a novel metric for topological reliability based on graph theory and integrates uncertainty modeling to assess the economic implications under variable conditions.
- Section “Conclusion” summarizes the key findings, offers concluding remarks, and outlines potential avenues for future research.

## Methodology

**Fig. 1:**
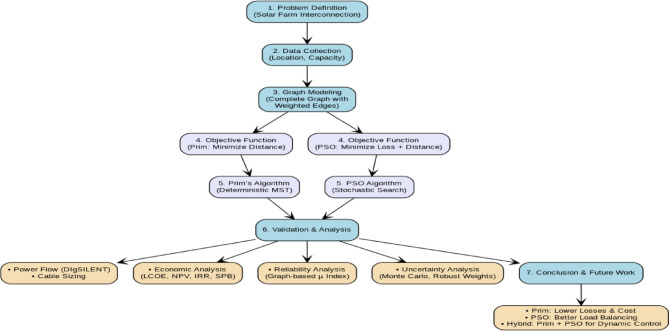
Methodological framework for optimized solar farm interconnection integrating prim’s and PSO algorithms.

This study aims to design an optimal network for interconnecting nine solar farms (each 2.5 MW) to an MV distribution substation, as outlined in Fig. 1. The core challenges addressed include minimizing energy losses, reducing capital expenditures, and enhancing system reliability all comprehensively analyzed throughout this work. The methodological workflow comprises: **(1) Data Collection** involving assumed geographical coordinates of solar farms (each with 2.5 MW nominal capacity) and an urban substation for grid injection; **(2) Graph Modeling** representing all feasible interconnection topologies; **(3) Objective Function Definition** where Prim’s algorithm minimizes total edge weights (path distances), while PSO is evaluated under two distinct objectives: active power loss minimization and path length minimization (cost reduction); **(4) Algorithm Implementation applying Prim’s** algorithm to construct a Minimum Spanning Tree (MST) based on distance weights yielding the shortest collective path; **(5) Algorithm Implementation applying PSO**’s intelligent algorithm search for Pareto-optimal solutions balancing loss/cost trade-offs; **(6) Validation and Analysis** through co-simulation in DigSILENT and Python; and **(7) Conclusions** synthesizing findings and proposing future research directions.

It should be noted that the current analysis exclusively utilizes nominal power parameters of solar farms, with production uncertainties excluded from the present workflow. However, we propose incorporating uncertainty scenarios—through statistical distributions or annual production/load data analysis in subsequent research. A brief examination of uncertainty impacts on economic parameters and reliability is included. Furthermore, the proposed framework is generalizable to arbitrary configurations and scales of hybrid energy resources (e.g., wind-solar-storage systems).

### Graph theory fundamentals

A graph is defined as an ordered pair (V(G), E(G)) where:

- V(G): Non-empty set of vertices (nodes).
- E(G): Set of edges disjoint from V(G), where each edge represents an unordered pair of distinct vertices.

**Fig. 2:**
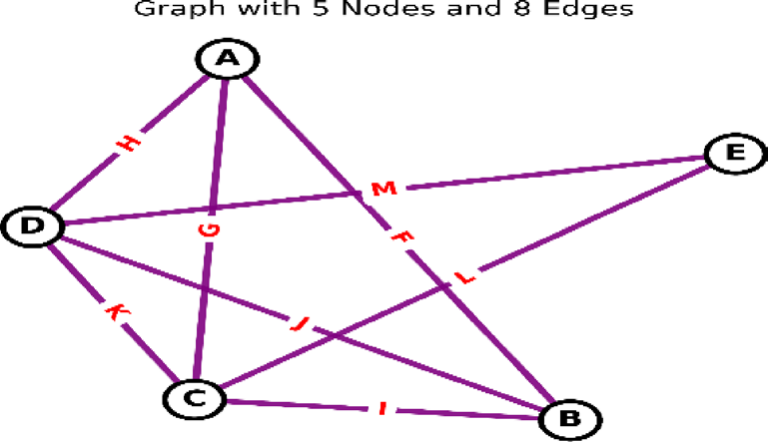
Undirected graph (5 nodes, 8 edges) and its spanning tree.

The number of vertices denotes the graph’s *order*, while the number of edges constitutes its *size*^61,62^. Graphs are classified as *undirected* when edges lack orientation, otherwise deemed *directed*. Figure 2 illustrates an undirected graph with 5 vertices and 8 edges.

#### Key graph terminology

**Fig. 3:**
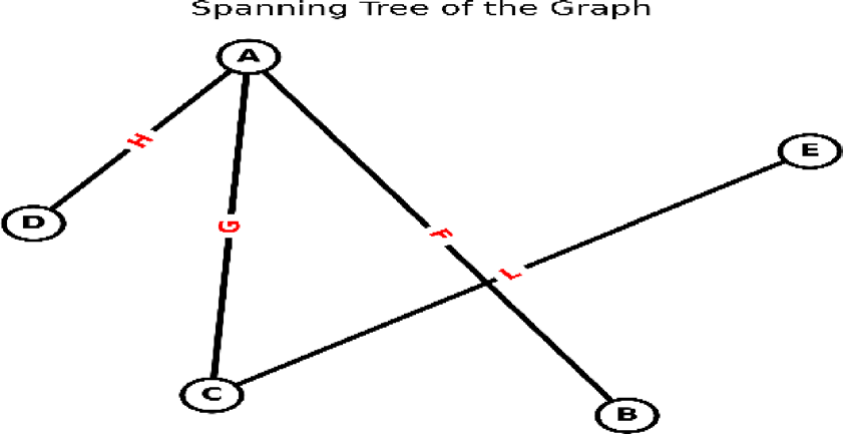
Spanning tree derived from Fig. 2.

- **Complete Graph**: A simple graph in which every pair of vertices is connected by a unique.
- **Tree**: Connected graph without cycles.
- **Spanning Tree**: A subgraph that: : A subgraph that (1) is a tree and (2) includes all vertices of the original graph (Fig. 3).

### Prim’s algorithm implementation

Prim’s algorithm is a greedy algorithm used to compute the Minimum Spanning Tree (MST) of a connected, weighted, undirected graph. It starts from an arbitrary node and iteratively adds the lowest-weight edge that connects any node in the growing tree to a node outside of it while avoiding two conditions: cycle formation, and disconnection (i.e., avoiding forest creation).At each step, the algorithm selects the next lightest edge available that maintains connectivity and acyclicity. The MST ultimately produced minimizes the total sum of edge weights offering the shortest possible connected structure among all nodes^63–65^.

### PSO algorithm

Particle Swarm Optimization (PSO) is a metaheuristic algorithm inspired by the collective behavior of swarms in nature, such as bird flocks and fish schools. Unlike deterministic graph-based approaches, PSO operates in continuous search space and does not guarantee global optimality, but it is valued for its flexibility, ease of implementation, and efficient convergence.

In PSO, a population of candidate solutions known as “particles” move through the solution space. Each particle adjusts its position based on: Its own experience (personal best/P-best), the global best experience among all particles (G-best).

During each iteration:

1. Particles update their positions and velocities using predefined mathematical equations (Eqs. 1–5).
2. The fitness value of each particle is calculated relative to the defined objective function.
3. Particles adjust their trajectories toward more promising areas in the solution space.

Depending on the optimization criteria (minimization or maximization), the best-performing particle is retained as the system moves toward convergence.

#### PSO formulation

The Particle Swarm Optimization (PSO) algorithm can be implemented based on Eq. 1 to 5 as follows:

1 $$\:\text{m}\text{i}\text{n}\:f\left(X\right)=\left({X}_{1},{X}_{2},\dots\:{X}_{n}\right)\:\:\:{X\epsilon\left[{{a}_{i},b}_{i\:}\right]}_{\:\:\:\:\:\:\:}$$

2 $$\:{X}_{i}^{t}\:=\:\left({X}_{i1},{X}_{i2},\dots\:{X}_{in}\right)$$

3 $$\:{V}_{i}^{t}\:=\:\left({V}_{i1},{V}_{i2},\dots\:{V}_{in}\right)$$

4 $$\:{V}_{i}^{t+1}=\text{w}\:{V}_{i}^{t}+{c}_{1}{r}_{1}\left(\:{P}_{i}-{X}_{i}^{t}\right)+{c}_{2}{r}_{2}\left(\:{P}_{g}-{X}_{i}^{t}\right)$$

5 $$\:{X}_{i}^{t+1}=\:{X}_{i}^{t}+{V}_{i}^{t+1}$$

where the position of the i-th particle at iteration t is represented by$\:{\:\:X}_{i}^{t}$, the position of the i-th particle at iteration t + 1 is denoted by $\:{X}_{i}^{t+1}$, the velocity of the i-th particle at iteration t is represented by $\:{V}_{i}^{t}$, and the velocity of the i-th particle at iteration t + 1 is denoted by $\:{V}_{i}^{t+1}$. Additionally, the personal best of the i-th particle (the best objective function value obtained by the particle up to iteration t) is represented by $\:{P}_{i}$, and the global best (the best objective function value obtained among all particles up to iteration t) is represented by$\:\:\:{P}_{g}$.Furthermore$\:\:{c}_{1}$,$\:{c}_{2}$ and are random numbers, and, typically set to 0.7 for convergence.

## System under study and prim’s algorithm implementation

To evaluate the proposed methodology, nine solar farms are hypothetically positioned at distinct geographical coordinates around a central distribution substation. Table 1 specifies the longitudinal and latitudinal coordinates of each farm’s transformer location, with each facility having a nominal generation capacity of 2.5 MW.

**Table 1:** Geospatial coordinates of solar farms and distribution Substation.

Geographical coordinates of the transformers location		
Solar Farm No.	Latitude	Longitude
0	31.87°	54.295°
1	31.92°	54.361°
2	31.95°	54.35°
3	31.9°	54.18°
4	31.896°	54.33°
5	31.77°	54.23°
6	31.84°	54.27°
7	31.82°	54.22°
8	31.8°	54.10°

Geographical coordinates of the substation **location**		
Substation No.	Latitude	Longitude
9	31.8972°	54.3678°

**Fig. 4:**
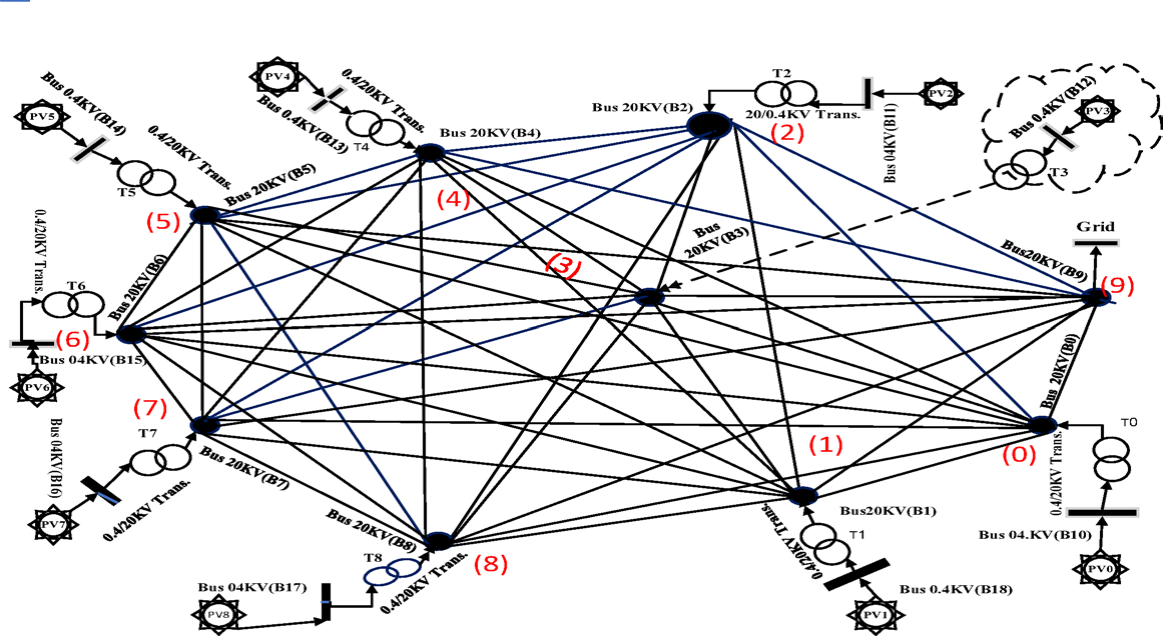
Completed graph of all possible connection states for 9 photovoltaic plants and a MV substation.

**Fig. 5:**
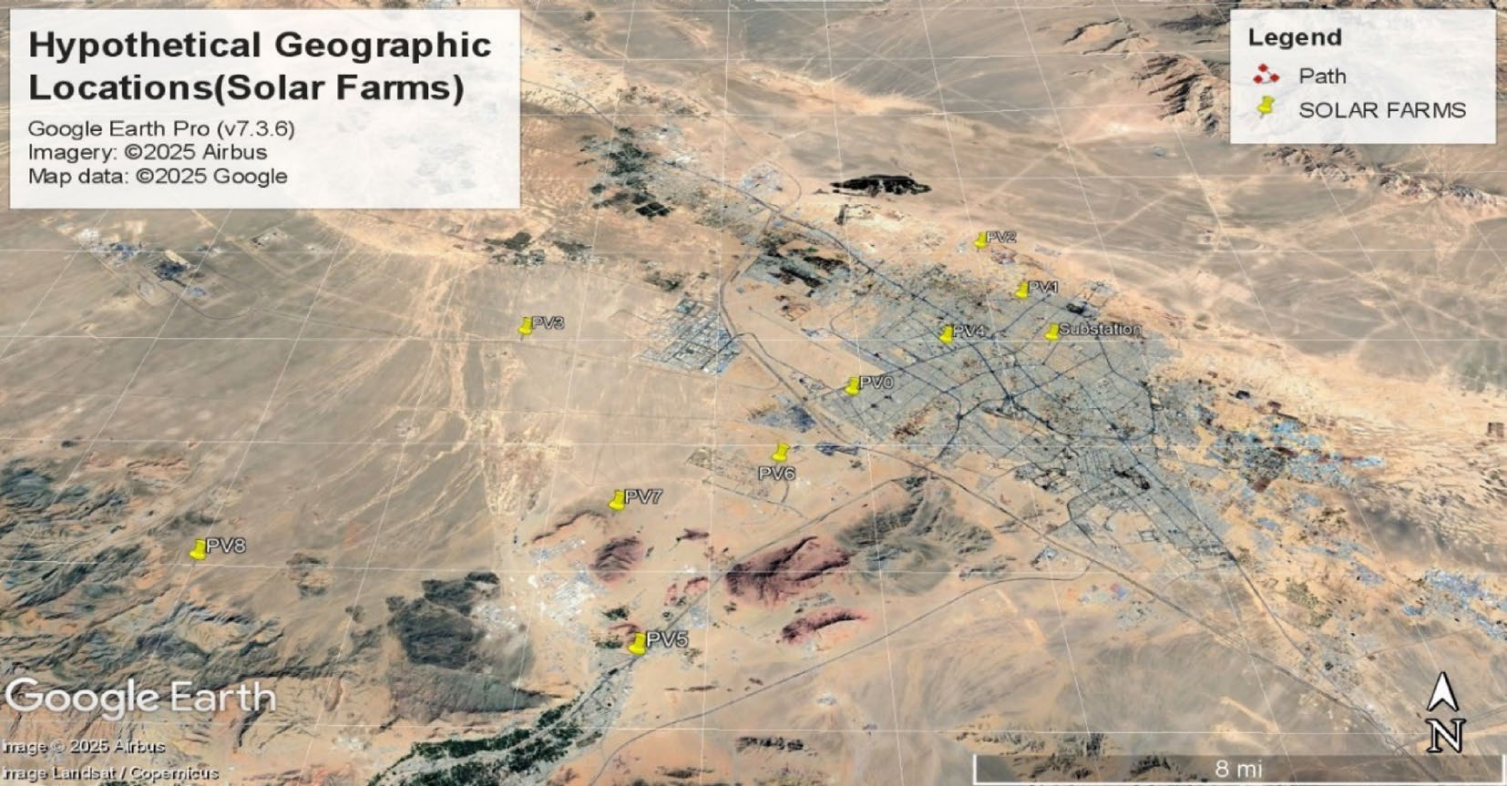
Hypothetical geographic positioning of PV arrays (solar farm inverter locations), Software: Google Earth Pro (v7.3.6), Imagery Date: 2025^66^, Imagery sources: Airbus, Landsat/Copernicus (2025)Map data: Google LLC (2025).

A complete graph topology encompassing all possible interconnections between the nine solar farms and the distribution substation (designated as GRID) was constructed. Figures 4 and 5 respectively illustrate:

- The exhaustive connection possibilities within the network.
- The actual geospatial layout of components^66^.

### Component labeling and optimization procedure

The system employs standardized nomenclature: 20 kV buses are labeled B0–B9, while 400 V buses are designated B10–B18. Solar farms appear as PV0–PV8, and 0.4/20 kV transformers as T0–T8. All potential 20 kV interconnections (totaling 45 lines, L50–L94) form a complete graph topology. Optimization algorithms analyze these connections to identify 9 lines constituting a spanning tree that optimally satisfies predefined objectives.

#### Distance calculation phase

Geodesic distances between solar farms and to the distribution substation (Table 1 coordinates) are computed as edge weights for the complete graph (Fig. 4). This weighted graph serves as input for Prim’s algorithm.

#### Five optimization scenarios

1. **Prim Base Case**: Direct application to the complete graph.(Case1).
2. **Prim Secondary**: Remove edges from Scenario 1’s spanning tree; reapply Prim to the residual graph. (Case2)
3. **Prim Tertiary**: Remove edges from Scenarios 1–2 results; reapply Prim to obtain a third spanning tree. (Case3)
4. **PSO Loss Minimization**: Extract optimal path minimizing total power losses. (Case4)
5. **PSO Cost Minimization**: Extract optimal path minimizing total line length. (Case5)

#### Visualization and validation

Resulting topologies from all five scenarios are illustrated in Figs. 6, 7, 8, 9, 10, 11, 12, 13, 14 and 15, depicting geographic interconnection schemes. Python-implemented Prim paths (Scenarios 1–3) are tabulated in Tables 2, 3 and 4. Subsequent DigSILENT simulations apply NF C13-200 standards to determine:

**Table 2:** Proposed path and edge weights (distances between lines in kilometers) in the first case.

Python 3.13	
First-Final Node	Length Line(Km)
2 − 1	9.70752202
3 − 2	6.97703404
9 − 3	4.08694049
2–4	6.24095439
0–5	3.57099642
2–6	9.09391754
8 − 7	3.49361783
0–8	2.61523609
5–9	4.39078955

**Table 3:** Proposed path and edge weights (distance between lines in kilometers) in the second case.

Python 3.13	
First-Final Node	Length Line(Km) Line(Km)
4 − 1	12.73224527
9 − 2	10.55623721
5 − 3	8.418942025
3–4	8.652925123
7 − 5	6.294192739
3–6	10.80474965
0–7	6.106703690
5–8	3.960355471
0–9	7.509631372

**Table 4:** Proposed routes and distances (in kilometers) between lines in the third case.

Python 3.3.2	
First-Final Node	Length Line(Km)
6 − 1	13.44393342
5 − 2	14.90502799
0–3	11.2138453
9 − 4	12.70275815
6 − 5	14.16749171
9 − 6	11.35880113
9 − 7	10.29967621
3–8	12.36772824
8–9	8.350668381

Optimal cable sizing, Line parameters(Results presented in Table 5 and 6).

**Table 5:** Optimal sizes and calculated parameters from load flow analysis of spanning trees obtained from the three analyzed cases.

Case Number	Line Number	Line Length ($\:Km)$	Loading (%)	Losses (kW)	Cable Size ($\:{\:\text{m}\text{m}}^{2})$	Average Cable Size ($\:{\:\text{m}\text{m}}^{2})$	Average Loading (%)	Total Lenght (Km)	Total Losses (kW)
Case1	53	4.086	85.63	149.65	400				
	55	4.390	89.24	103.76	240				
	59	2.615	91.25	15.29	70				
	66	3.493	78.14	5.098	35				
	76	9.093	77.89	13.18	35	137	83.85	50.177	569.554
	80	3.570	88.10	97.04	150				
	87	6.240	78.01	9.077	35				
	91	6.977	88.55	162.36	240				
	93	9.707	77.87	14.067	35				
	50	7.509	90.012	646.21	800				
	52	10.55	79.79	16.06	35				
	94	3.960	75.25	5.359	35				
	67	6.106	88.42	397.78	630				
Case 2	72	6.294	86.61	298.47	500	264	81.82	75.03	1613.76
	77	10.804	73.74	14.079	35				
	83	8.4180	83.68	174.91	240				
	86	12.732	73.18	16.296	35				
	88	8.652	85.63	44.561	70				
	54	12.702	79.703	19.283	35				
	56	11.357	88.08	261.470	240				
	57	10.229	79.81	15.569	35				
	58	8.350	88.186	110.355	150				
Case 3	62	12.367	74.432	71.882	95	85	81.38	108.73	615.78
	75	13.443	77.895	19.493	35				
	79	14.167	89.867	80.352	70				
	82	14.904	76.735	20.973	35				
	89	11.213	77.747	16.198	70				

**Table 6:** Calculated parameters from load flow analysis for the proposed path of the PSO algorithm for two objective functions.

Case. Number	Line Number	Line Length (Km)	Loading (%)	Losses (kW)	Cable Size $\:{(\text{m}\text{m}}^{2})$	Average Cable Size ($\:{\:\text{m}\text{m}}^{2})$	Average Loading (%)	Total Length (Km)	Total Losses (kW)
Case 4	54	3.5709	91.830	318.329	400				
	63	24.205	94.465	508.03666	120				
	64	12.732	81.718	147.904	95				
	71	14.905	85.782	21.995	35				
	79	8.6529	5.54341	0.000234	35	93	76.48	115.49	1153.057
	82	23.00	83.019	116.92	50				
	86	3.9603	86.284	5.9129	35				
	88	14.167	85.816	20.923	35				
	92	10.299	79.416	13.027	35				
Case 5	51	2.6152	89.90635	223.46	400				
	59	19.998	87.77607	631.43	185				
	64	12.732	92.08231	253.918	120				
	72	6.2409	79.50534	7.9112	35				
	77	10.804	99.12031	52.831	35	153	79.58	86.375	1691.29
	79	8.6529	99.12031-0.	42.309	35				
	85	4.3907	90.07654	203.916	240				
	86	3.96035	90.8955	254.91	300				
	91	16.97988	77.77845	20.599	35				

**Fig. 6:**
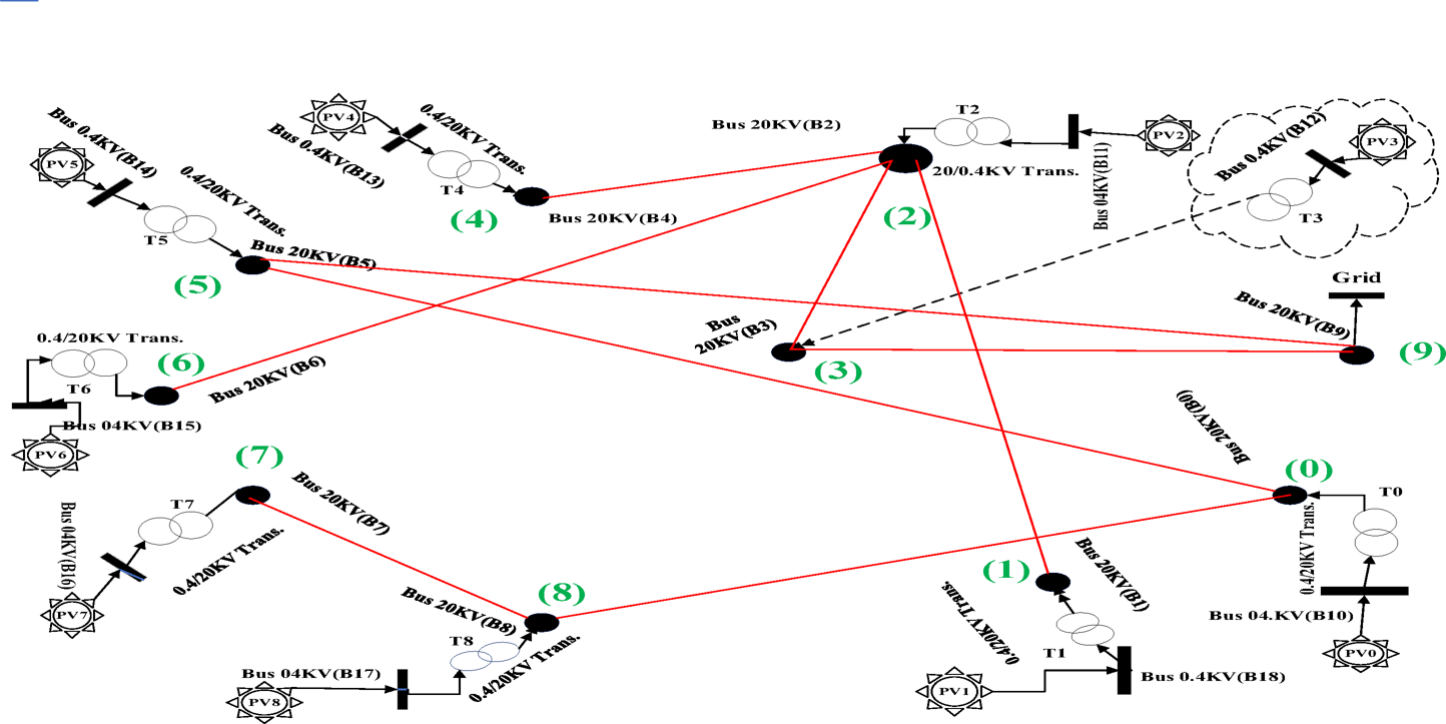
Spanning tree in the first case with 10 nodes, including 9 solar farms and one MV substation.

**Fig. 7:**
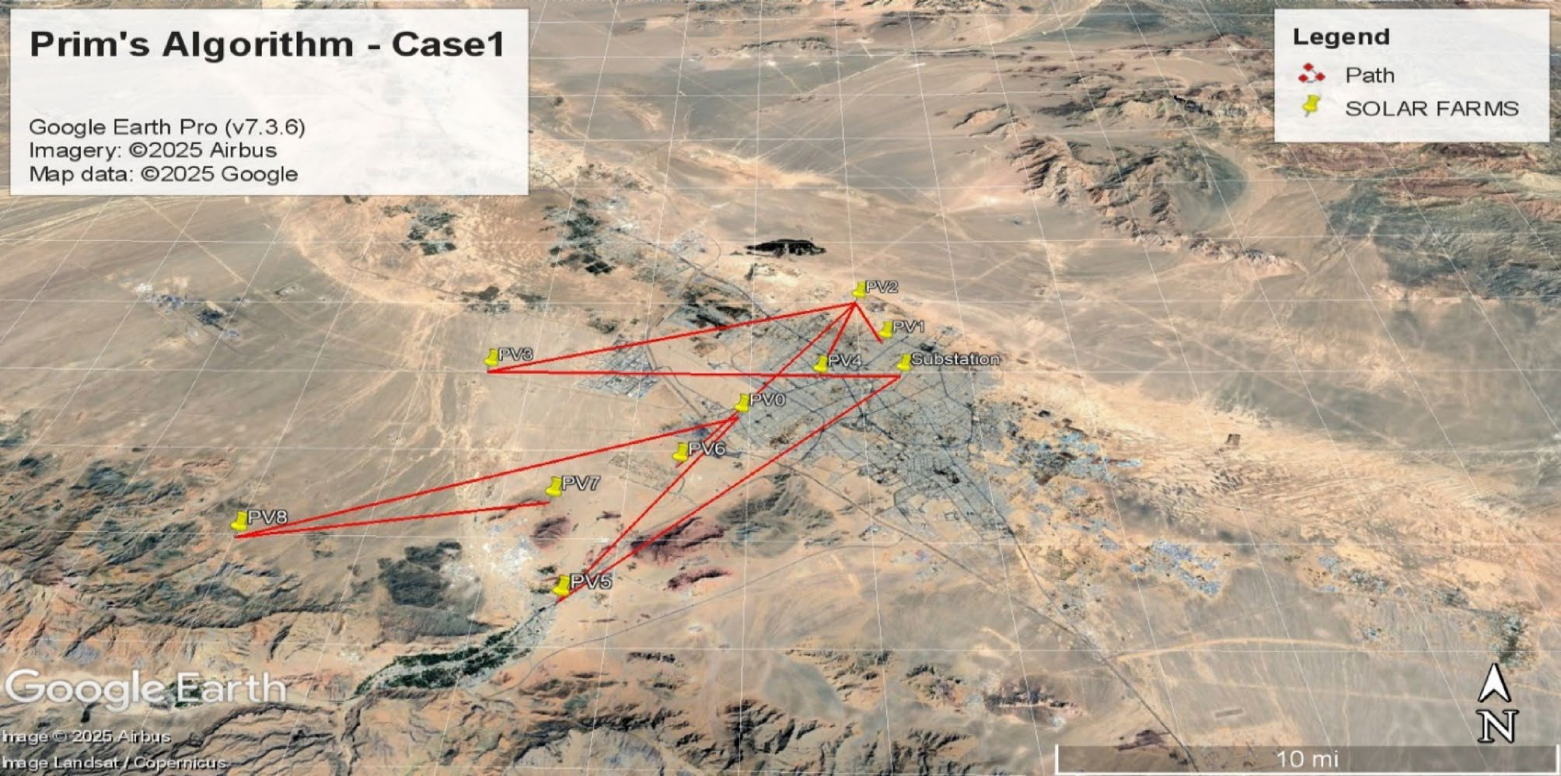
Geographic Layout and Interconnection Topology Based on Table 2 Paths (Prim’s Algorithm - Case1). Software: Google Earth Pro (v7.3.6), Imagery Date: 2025^66^, Imagery sources: Airbus, Landsat/Copernicus (2025). Map data: Google LLC (2025).

**Fig. 8:**
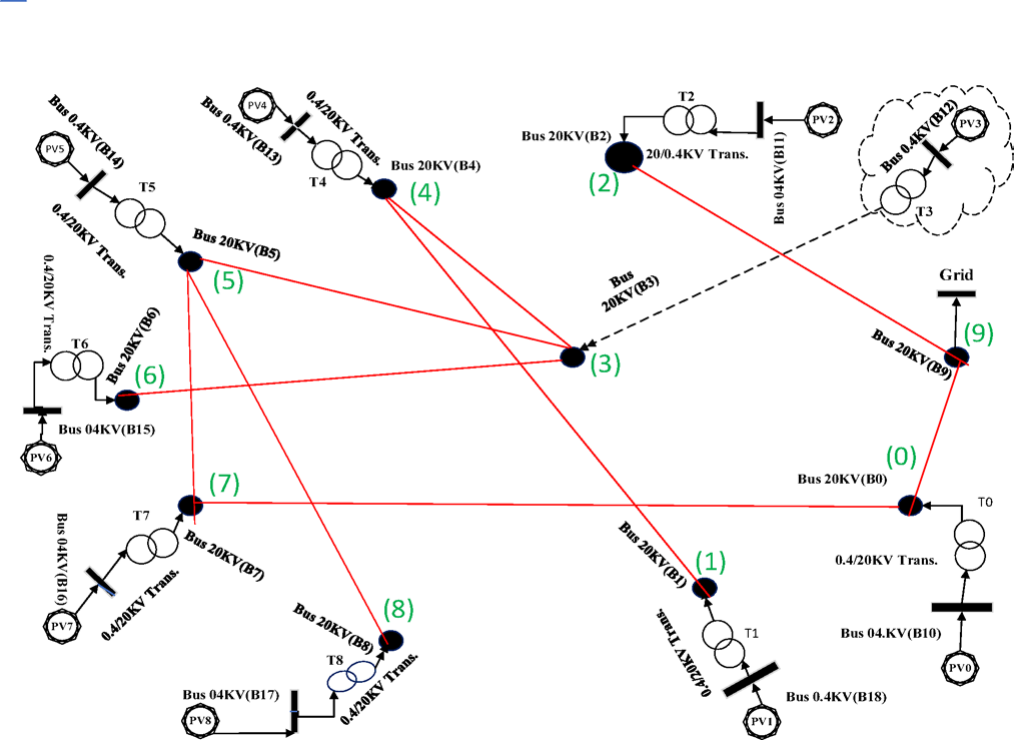
Spanning tree in the second case with 10 nodes including 9 solar farms and one MV substation.

**Fig. 9:**
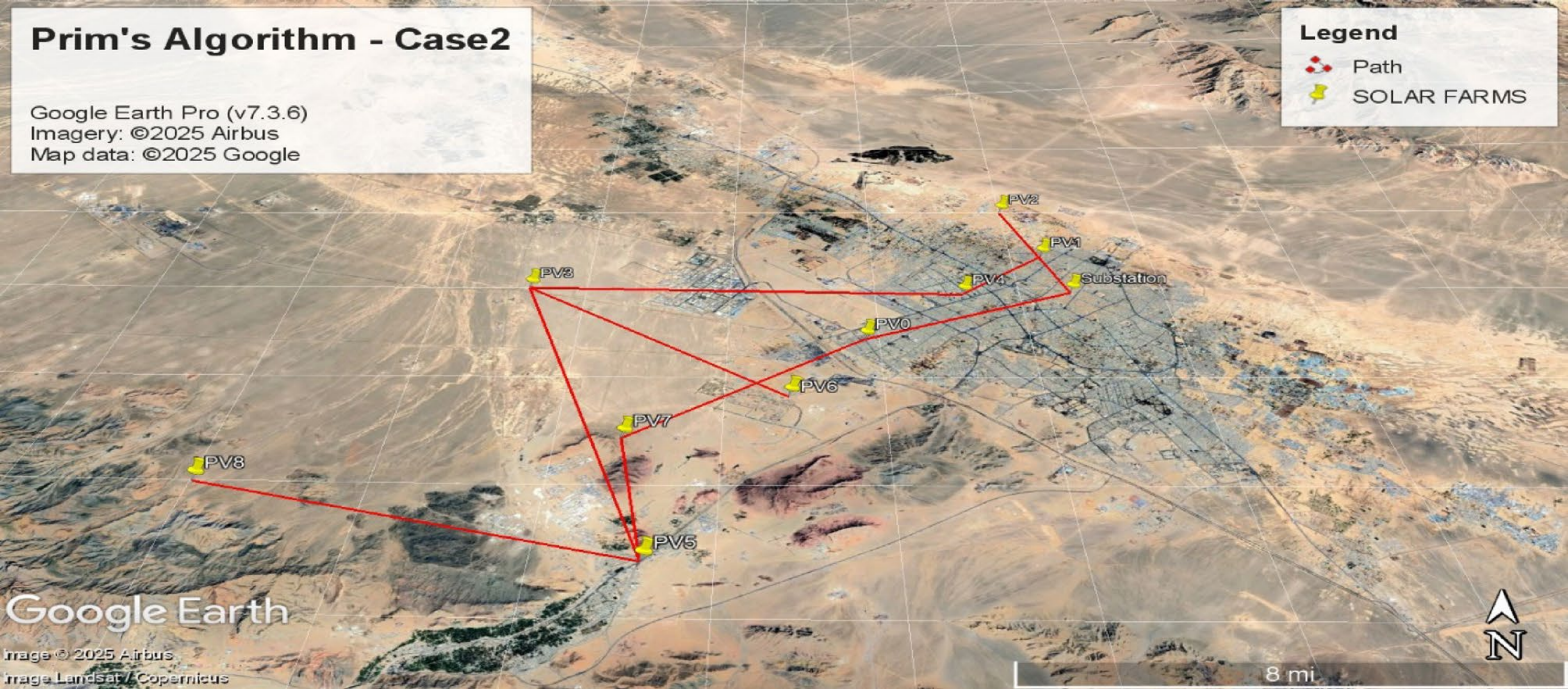
Geographic Layout and Interconnection Topology Based on Table 3 Paths (Prim’s Algorithm – Case2). Software: Google Earth Pro (v7.3.6), Imagery Date: 2025^66^, Imagery sources: Airbus, Landsat/Copernicus (2025),Map data: Google LLC (2025).

**Fig. 10:**
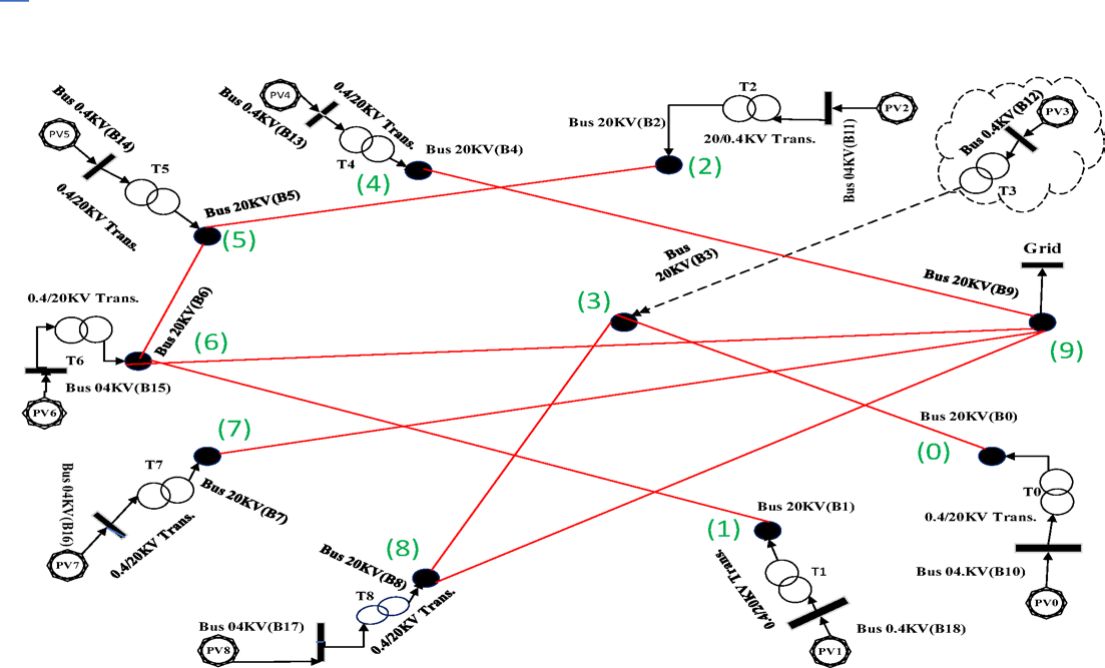
Spanning tree for the third case with 10 nodes, including 9 solar farms and one MV substation.

**Fig. 11:**
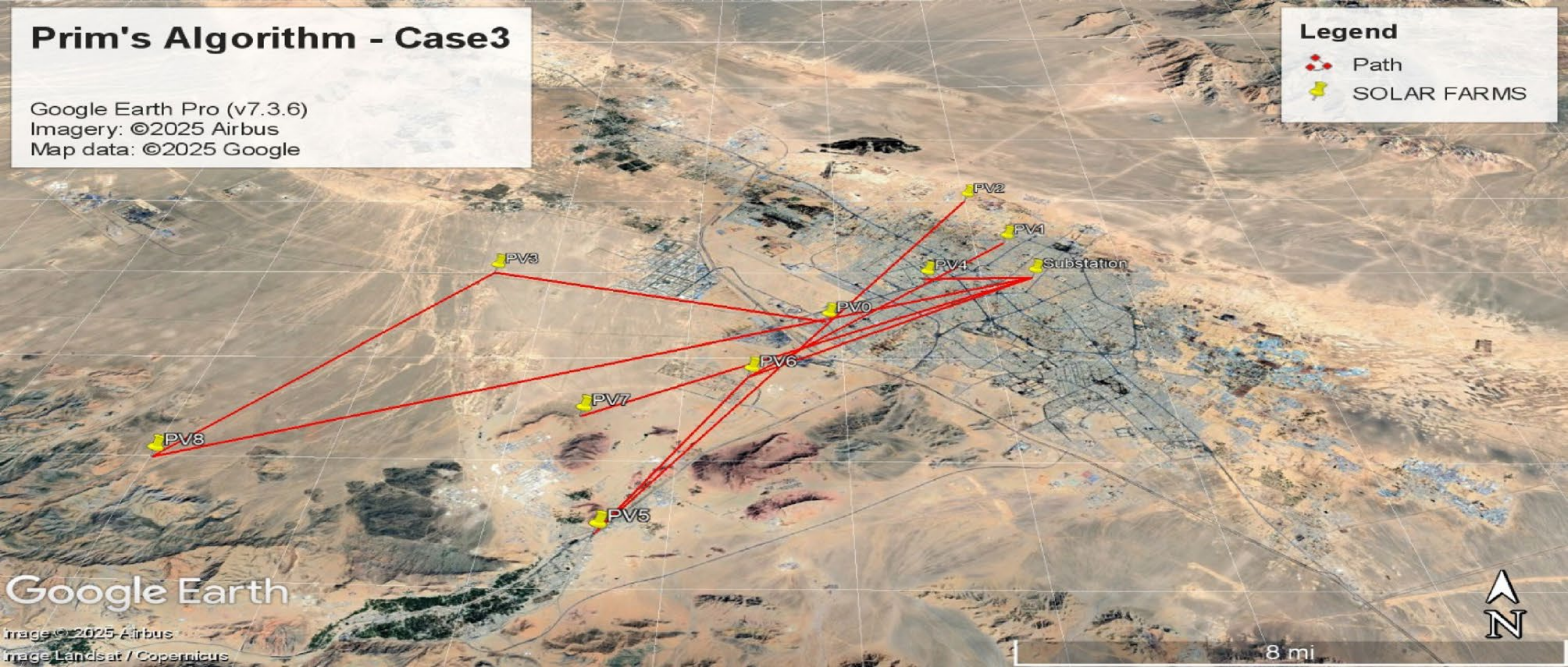
Geographic Layout and Interconnection Topology Based on Table 3 Paths (Prim’s Algorithm – Case3). Software: Google Earth Pro (v7.3.6), Imagery Date: 2025^66^, Imagery sources: Airbus, Landsat/Copernicus (2025),Map data: Google LLC (2025).

### PSO algorithm implementation

The PSO algorithm was implemented using the mathematical formulation outlined in Sect. 2.3. The algorithm was executed in Python, integrated within the DIgSILENT PowerFactory simulation environment. Two distinct objective function scenarios were considered: Minimizing the total line losses, and minimizing the total line length.It is important to clarify a key limitation regarding Prim’s algorithm in this context: Prim’s algorithm cannot be used when the objective function is total power loss. This restriction arises from the way data is exchanged between Python and DIgSILENT. Specifically, the path length between any two solar farms remains constant within each simulation and can be treated as a fixed edge weight in the graph. However, power losses are dependent on the actual network topology and vary with the configuration of the graph. Therefore, power loss values computed from load flow analysis on one configuration (e.g., a complete graph) cannot be generalized as edge weights for other configurations.As a consequence, it is not feasible to use Prim’s algorithm to construct minimum spanning trees in Cases 1–3 when power loss serves as the objective function. In contrast, the PSO algorithm does not rely on fixed edge weights. Instead, PSO evaluates each candidate topology dynamically by running load flow calculations for every configuration, enabling it to directly optimize for loss or length in a flexible, topology-dependent manner.The fundamental distinction between Prim’s and PSO algorithms lies in their execution style and outcomes:

Prim’s algorithm is a deterministic method that generates a unique Minimum Spanning Tree (MST). Once the MST is established, subsequent optimization (e.g., cable sizing) can be performed based on detailed power system parameters including line losses, line loading, and power flow calculations.PSO, on the other hand, is a stochastic optimization technique. Due to its reliance on random number generation, it does not produce a single deterministic solution. However, by increasing the number of iterations and particles, the algorithm can converge toward a near-optimal solution with high accuracy.The convergence behavior of the PSO algorithm was tracked and is illustrated in Fig. 16. Additionally, simulation run-times for various test scenarios are summarized in Table 7.

**Table 7:** Simulation time comparison between algorithms.

Network Size (Number of Solar Farms)	Algorithm Used	Average Simulation Time (seconds)	Convergence Speed	Notes
9	Prim	2.1	Fast	Graph expansion becomes too complex
9	PSO(Path Losses)	480–600	Slow, Accepted	Heuristic approach required
9	PSO(Path Length)	180–240	Slow, Accepted	Heuristic approach required

**Fig. 12:**
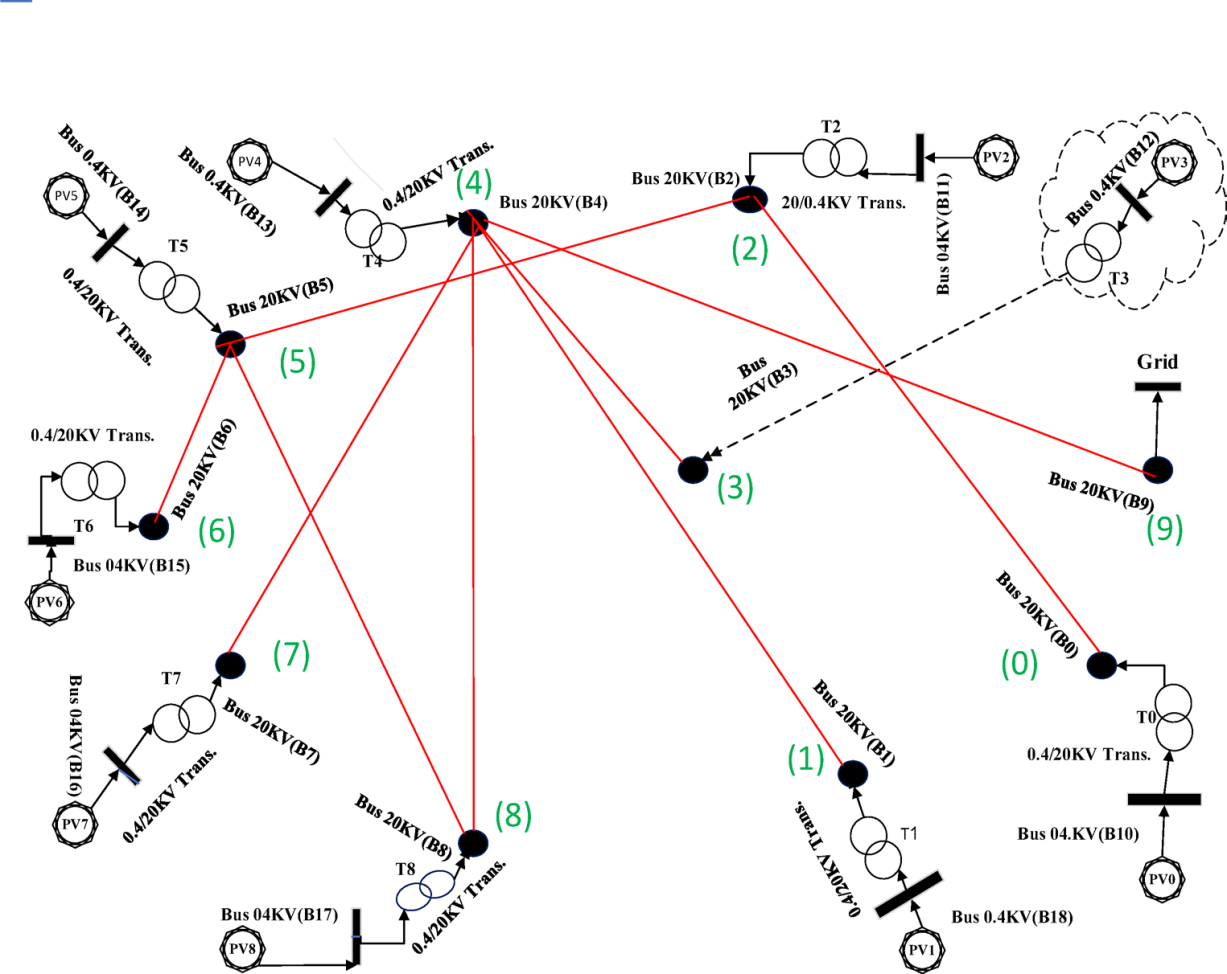
Spanning tree for the fourth case with 10 nodes including 9 solar farms and one MV substation, obtained using the PSO algorithm with the objective of minimizing losses(Case 4).

**Fig. 13:**
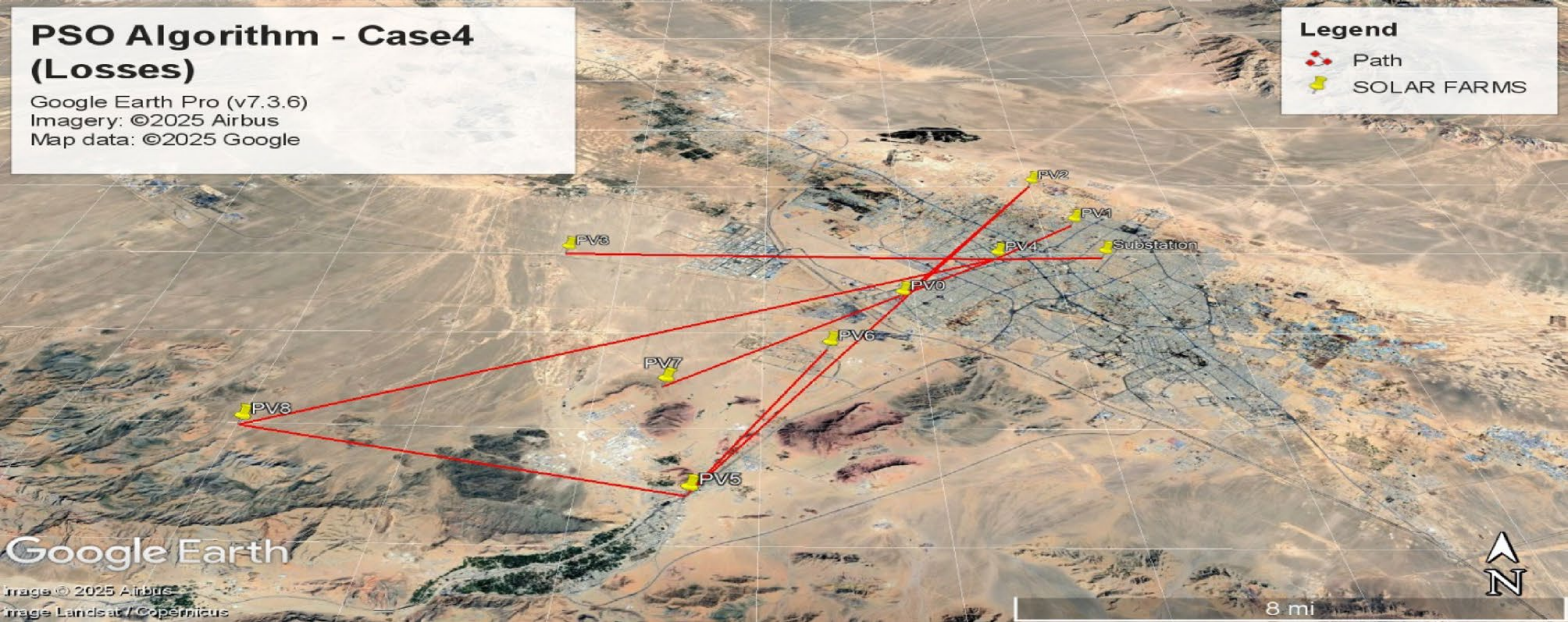
Geographic Layout and Interconnection Topology Based on Fig. 12 Paths (PSO Algorithm – Case4). Software: Google Earth Pro (v7.3.6), Imagery Date: 2025^66^, Imagery sources: Airbus, Landsat/Copernicus (2025),Map data: Google LLC (2025).

**Fig. 14:**
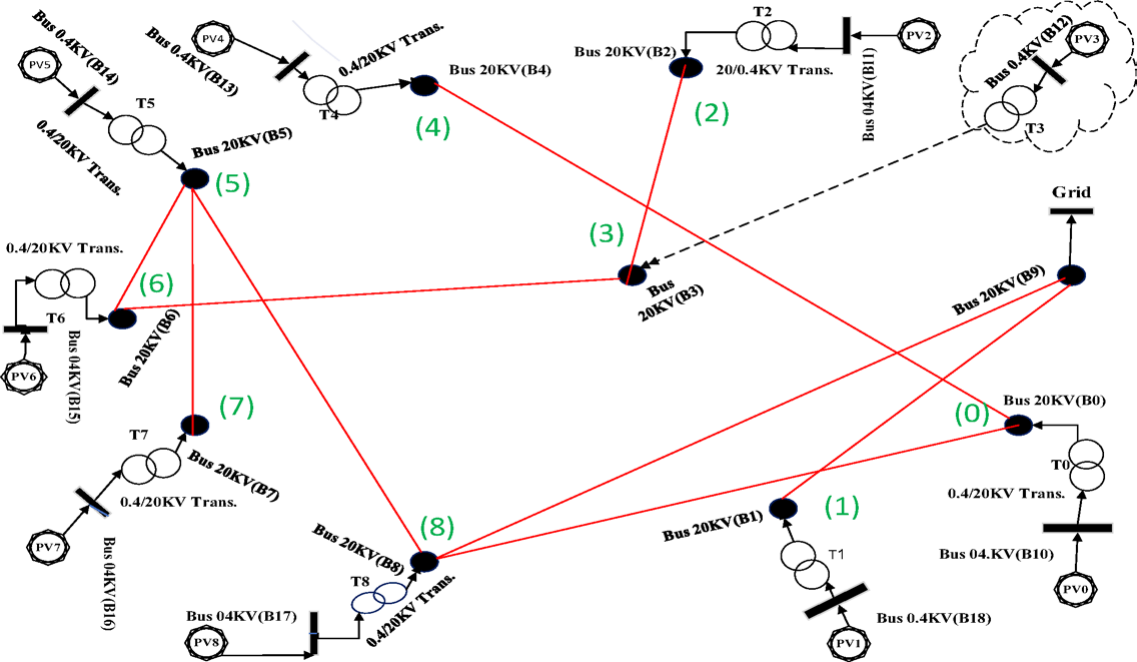
Spanning tree for the fifth case with 10 nodes including 9 solar farms and one MV substation, obtained using the PSO algorithm with the objective of minimizing path length(Case 5).

**Fig. 15:**
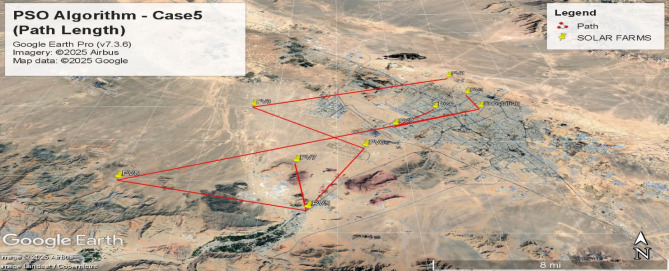
Geographic Layout and Interconnection Topology Based on Fig. 14 Paths (PSO Algorithm – Case5). Software: Google Earth Pro (v7.3.6), Imagery Date: 2025^66^, Imagery sources: Airbus, Landsat/Copernicus (2025),Map data: Google LLC (2025).

Figures 12, 13, 14 and 15 visualize the interconnection paths generated under different PSO parameter settings, highlighting variations in network formation and node connectivity.

#### PSO parameters

Swarm size : 50 particles, Maximum iterations : 100,Network configuration : 10 nodes (9 solar farms + 1 substation), Complete graph edges : 45 connections.

#### Power loss optimization (Case4-Bottom Plot)

#### Path length optimization (Case 5 - Bottom Plot)

#### Convergence(plot in Fig. 16)

- Demonstrates a reduction in total losses from an initial value of **1.15 MW** to a final value of **1**,**153.057 kW**,Achieves stable convergence around **iteration 60**,final loss represents a **0.057% improvement** over the baseline value(Case4).
- Converges to the target total path length of **86.375 km** by approximately **iteration 20**,shows consistent improvement in route efficiency, Maintains less than **0.01% variation** after convergence.(Case5).

These results validate that:

- **Both objective functions** (loss and length minimization) reach stable solutions within **100 iterations**.
- The selected PSO parameters (**w = 0.7**,** c1 = c2 = 1.49**) provide a good balance between **exploration and exploitation**.
- There is **no premature convergence**, indicating robust algorithm performance.

**Fig. 16:**
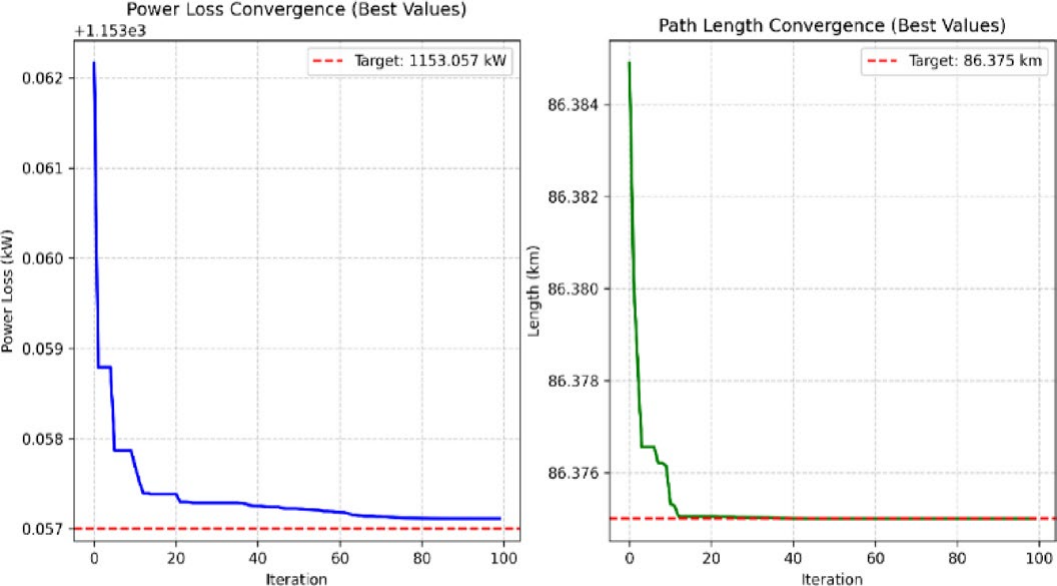
Convergence plot for PSO algorithm(Case 4,Case 5).

### Load flow analysis and comparative discussion

A comparative analysis of load flow results, as presented in Table 5, reveals that Case 1 based on the topology generated by Prim’s algorithm demonstrates both the shortest total path length and the lowest power losses among all evaluated configurations. Although this outcome aligns with expectations, Cases 2 and 3 were intentionally derived by excluding previously optimal paths, to explore secondary and tertiary routing strategies. This approach allows for the assessment of alternative feasible paths and their potential impact on power loss mitigation and network reliability, both of which are discussed further in subsequent sections.A fundamental limitation of Prim’s algorithm is that it relies solely on fixed edge weights, typically based on physical path length. This design prevents the algorithm from accounting for dynamic variables such as real-time losses or reliability factors within the optimization process. Since power losses vary based on network topology, loss values obtained from a load flow analysis on a complete graph cannot be reused or applied as edge weights in alternate topologies. Consequently, it is not feasible to use Prim’s algorithm to construct minimum-loss spanning trees, making it unsuitable for cases where power loss is the primary optimization criterion. However, an alternative edge-weighting strategy to address this limitation is introduced in the uncertainty analysis section of this paper.An interesting observation is that the total power loss in Case 2 is actually higher than in Case 3, despite Case 2 displaying a seemingly more compact path configuration. This outcome supports the theory that excluding optimal paths often leads to suboptimal performance, emphasizing the importance of multi-objective trade-off analysis. The improved performance in Case 3 can be attributed to the nonlinear relationship of power losses with system parameters. Specifically, power losses are directly proportional to the line length and the square of the current, but inversely proportional to the cable cross-sectional area. Although Case 3 involves a longer path and smaller average cable sizing, the significant reduction in current (and therefore in current squared) effectively compensates for those drawbacks, resulting in overall lower losses.From Table 5, it is evident that the network generated via Prim’s algorithm in terms of both path efficiency and electrical performance results in the most optimal configuration when nominal weights are assumed to reflect distance.In real-world scenarios, natural obstacles such as mountains, forests, or bodies of water may impose restrictions on feasible interconnection paths. To simulate such geographic limitations in the modeling phase, artificially large weights (e.g., ten times the longest feasible edge) can be assigned to these restricted routes, effectively excluding them from path selection during algorithm execution. In this study, however, it was assumed that all solar farms are located within an open and unobstructed desert region, enabling direct application of Prim’s algorithm to the complete graph.

#### Insights from Tables 5 and 6

The comparison between Tables 5 and 6 supports several noteworthy conclusions:

##### Average line loading

The PSO configuration optimized for minimizing power loss (Case 4) exhibits the lowest average line loading among all evaluated cases, including Case 1 (Prim’s) and Case 5 (PSO optimized for path length).

Green zone: loading below 80%,Yellow zone: 80–100%,Red zone: above 100% (overloaded).

In Case 4, Line No. 79 shows a loading below 10%, which notably reduces the overall average. However, since none of the configurations breached the red zone, all proposed networks are deemed acceptable under nominal loading conditions. Interestingly, Prim’s algorithm results in a greater number of lines operating within the green zone, suggesting that it provides more balanced and conservative line utilization compared to the PSO-based solutions.

##### Total power losses

###### Case 1

(Prim’s algorithm) yields the lowest total power losses relative to both Cases 4 and 5 (PSO-based topologies). This reinforces the practicality of deterministic, graph-theoretic approaches particularly in large-scale renewable deployments where minimizing operational losses and capital costs is crucial.

#### PSO case comparison

Within the PSO-based scenarios:

##### • Case 4

(loss minimization objective) successfully reduces power losses more than Case 5.

##### • Case 5

(path length minimization) achieves the shortest cumulative distance.

As expected, each PSO configuration meets its respective goal. However, when compared to the Prim-based design, Case 1 still outperforms both in terms of total losses and practical feasibility, suggesting that deterministic methods remain highly competitive or even preferable in structured planning environments.

#### Algorithmic characteristics

- **Prim’s Algorithm**:

Operates under a deterministic framework, leveraging fixed edge weights (e.g., physical distances). It is best suited when a singular, well-defined metric (like path length) is the basis of optimization. It is computationally fast and produces unique, reproducible solutions.

- **Particle Swarm Optimization:**

As a stochastic, population-based method, PSO is capable of addressing a broader range of flexible and multi-objective functions, including those involving dynamic or topology-dependent variables such as power losses, reliability, or cost. While it does not guarantee a global optimum, it is highly adaptable and can approach near-optimal solutions through iterative improvements.

## Economic Estimation and reliability analysis

### Cost Estimation of paths identified by the algorithms

Building upon the results from previous sections and using the power flow data presented in Tables 8 and 9 an economic evaluation has been conducted for the transmission paths identified by both Prim’s algorithm and the PSO method.

**Table 8:** Approximate cost estimates for the spanning tree obtained from prim’s algorithm (cases 1 to 3).

(1) Case No. ($$\:\text{\$}$$/m)	(2) Line No.	(3) Cable Size ($$\:{\:\text{m}\text{m}}^{2})$$	(4) Line Length (km)	(5) Average Cable Price ($$\:\text{\$}$$/m)	(6) No. Phase	(7) Sum Cables Prices ($)	(8) Dimation Channel Excavation ($$\:{\:\text{m}}^{2})$$	(9) Total Line Length (km)	(10) Price Excavation &Fillig ($/$$\:{\:\text{m}}^{3}$$)	(11) Total Prices Excavation ($)	(12) Total Costs ($)
Case1	53	400	4.086	45	3	551,610	0.36	50.171	3	54,191	2,172,741
	55	240	4.390	28	3	368,760					
	59	70	2.615	7.9	3	61,975					
	66	35	3.493	4.3	3	45,059					
	76	35	9.093	4.3	3	117,299					
	80	150	3.570	17	3	182,070					
	87	35	6.240	4.3	3	80,496					
	91	240	6.977	28	3	586,068					
	93	35	9.707	4.3	3	125,220					
	Total cables Prices($)					2,118,557					
	50	800	7.509	84	3	1,892,268	0.36				
	52	35	10.55	4.3	3	136,095					
	94	35	3.960	4.3	3	51,084					
	67	630	6.106	73	3	1,337,214					
Case2	72	500	6.294	56	3	1,057,392					
	77	35	10.804	4.3	3	139,371		75.03	3	81,032	5,763,223
	83	240	8.4180	28	3	707,112					
	86	35	12.732	4.3	3	156,603					
	88	70	8.652	7.9	3	205,052					
	Total cables Prices($)					5,682,191					
	54	35	12.702	4.3	3	163,855	0.36				
	56	240	11.357	28	3	953,988					
	57	35	10.229	4.3	3	131,954					
	58	150	8.350	17	3	425,850					
Case3	62	95	12.367	11	3	408,111					
	75	35	13.443	4.3	3	173,414		108.73	3	117,428	3,003,564
	79	70	14.167	7.9	3	335,756					
	82	35	14.904	4.3	3	192,261					
	89	70	11.213	4.3	3	100,917					
	Total cables Prices($)					2,886,136					

**Table 9:** Approximate cost estimates for the spanning tree obtained from the PSO algorithm (cases 4 and 5).

(1) Case No. ($$\:\text{\$}$$/m)	(2) Line No.	(3) Cable Size ($$\:{\:\text{m}\text{m}}^{2})$$	(4) Line Length (km)	(5) Average Cable Price ($$\:\text{\$}$$/m)	(6) No. Phase	(7) Sum Cables Prices ($)	(8) Dimation Channel Excavation ($$\:{\:\text{m}}^{2})$$	(9) Total Line Length (km)	(10) Price Excavation &Fillig ($/$$\:{\:\text{m}}^{3}$$)	(11) Total Prices Excavation ($)	(12) Total Costs ($)
Case 4	54	400	3.57	45	3	481,950	0.36	115.43	3	124,664	3,004,618
	63	120	24.2	13.7	3	994,620					
	64	95	12.7	11	3	419,100					
	71	35	14.9	4.3	3	192,210					
	79	35	8.65	4.3	3	111,585					
	82	50	23	6	3	414,000					
	86	35	3.96	4.3	3	51,084					
	88	35	14.16	4.3	3	182,664					
	92	35	10.29	4.3	3	132,741					
	Total cables Prices($)					2,595,480					
Case 5	51	400	2.6152	45	3	353,025	0.36	86.375	3	93,285	3,238,813
	59	185	19.998	15.7	3	941,529					
	64	120	12.732	13.7	3	523,203					
	72	35	6.2409	4.3	3	80,507					
	77	35	10.804	4.3	3	139,372					
	79	35	8.6529	4.3	3	111,623					
	85	240	4.3907	28	3	368,819					
	86	300	3.96035	34.375	3	408,411					
	91	35	16.97988	4.3	3	219,039					
	Total cables Prices($)					3,145,528

- The cost estimation framework incorporates global average pricing for different cable cross-sections (see Column 7), covering both procurement and installation (burial) costs (see Column 11). The overall project implementation cost is summarized in Column 12.
- The cable purchase cost is computed based on the cable length, the number of phases, and the unit cost per phase (Columns 4–6), with results reported in Column 7. The installation cost, covering trenching, filling, and cable laying, is derived from trench diameter, line length, and excavation unit prices (Columns 8–10), and appears in Column 11. The total implementation cost — defined as the sum of material and installation costs — is presented in Column 12.

As can be observed, the minimum spanning tree generated via Prim’s algorithm applied to the complete graph (Case 1) results in the lowest total implementation cost. This cost is notably lower than those of other configurations: both the alternative Prim-based variations (Cases 2 and 3) and the PSO-generated networks (Cases 4 and 5). Thus, Case 1 proves to be the most cost-effective, achieving the lowest network losses and offering the least total installation cost making it a preferred solution from both economic and technical perspectives.

### Total cost Estimation metrics

Before comparing total project costs across different configurations, it is essential to define the key economic indicators and formulas used in this analysis:

1. **Annual Energy Production (AEP)**

*AEP* quantifies the total energy output from the solar farms over one year, considering rated capacity, annual operating hours, capacity factor, and energy losses:

7 $$\:AEP=\sum\:(Rated\:Capacity\left(MW\right)\text{*}Annual\:Hours\text{*}Capacity\:Factor$$

2. **Net Present Value (NPV)**

*NPV* represents the difference between discounted benefits and initial capital investment over a 20-year lifetime:

8 $$\:\text{N}\text{P}\text{V}=-\text{C}\text{A}\text{P}\text{E}\text{X}+{\sum\:}_{t=1}^{20}\frac{{Cash\:Flow}_{t}\:}{{\left(1+r\right)}^{t}\:}+\frac{\text{S}\text{a}\text{l}\text{v}\text{a}\text{g}\text{e}\:\text{V}\text{a}\text{l}\text{u}\text{e}\:\:}{{\left(1+r\right)}^{20}\:}$$

Where:

9 $$\:\text{C}\text{a}\text{s}\text{h}\:\text{F}\text{l}\text{o}\text{w}\:=\:(\text{A}\text{E}\text{P}\:-\:\text{L}\text{o}\text{s}\text{s}\text{e}\text{s})\:\times\:\:\text{E}\text{n}\text{e}\text{r}\text{g}\text{y}\:\text{P}\text{r}\text{i}\text{c}\text{e}\:-\:\text{O}\text{P}\text{E}\text{X}$$

- ***CAPEX***: Total upfront capital cost.
- ***OPEX***: Annual operation and maintenance cost.
- ***Salvage Value***: Residual asset value at the end of the project.
- ***r***: Discount rate (10%).
- ***n***: Project life (20 years).

3. **Total Net Present Cost (TNPC)**

10 $$\:\text{T}\text{N}\text{P}\text{C}\hspace{0.17em}=\hspace{0.17em}\text{C}\text{A}\text{P}\text{E}\text{X}\:+\:(\text{O}\text{P}\text{E}\text{X}\:\times\:\:\text{P}\text{V}\:\text{F}\text{a}\text{c}\text{t}\text{o}\text{r})\:-\:\text{S}\text{a}\text{l}\text{v}\text{a}\text{g}\text{e}\:\text{V}\text{a}\text{l}\text{u}\text{e}$$

4. **Levelized Cost of Energy (LCOE)**

*LCOE* expresses the average unit cost of electricity over the system’s life, considering discounted total costs and energy output:

11 $$\:\text{L}\text{C}\text{O}\text{E}\:\left(\frac{\text{\$}}{\text{k}\text{W}\text{h}}\right)=\frac{\left(\text{T}\text{o}\text{t}\text{a}\text{l}\:\text{D}\text{i}\text{s}\text{c}\text{o}\text{u}\text{n}\text{t}\text{e}\text{d}\:\text{C}\text{o}\text{s}\text{t}\text{s}\right)}{\left(\text{T}\text{o}\text{t}\text{a}\text{l}\:\text{D}\text{i}\text{s}\text{c}\text{o}\text{u}\text{n}\text{t}\text{e}\text{d}\:\text{E}\text{n}\text{e}\text{r}\text{g}\text{y}\:\text{P}\text{r}\text{o}\text{d}\text{u}\text{c}\text{t}\text{i}\text{o}\text{n}\right)}=\frac{{\sum\:}_{t=0}^{n}\left(\frac{Costs}{{\left(1+r\right)}^{t}}\right)}{{\sum\:}_{t=1}^{n}\left(\frac{Energ{y}_{t}}{{\left(1+r\right)}^{t}}\right)}\:\:$$

Supporting calculations:

12 $$\:\text{T}\text{o}\text{t}\text{a}\text{l}\:\text{D}\text{i}\text{s}\text{c}\text{o}\text{u}\text{n}\text{t}\text{e}\text{d}\:\text{E}\text{n}\text{e}\text{r}\text{g}\text{y}\:\text{P}\text{r}\text{o}\text{d}\text{u}\text{c}\text{t}\text{i}\text{o}\text{n}=\:\text{A}\text{n}\text{n}\text{u}\text{a}\text{l}\:\text{N}\text{e}\text{t}\:\text{P}\text{r}\text{o}\text{d}\text{u}\text{c}\text{t}\text{i}\text{o}\text{n}\text{*}\left(\frac{1-{\left(1+r\right)}^{-n}}{r}\right)$$

13 $$\:{PV}_{OPEX}=\text{A}\text{n}\text{n}\text{u}\text{a}\text{l}\:\text{O}\text{P}\text{E}\text{X}*\left(\frac{1-{\left(1+r\right)}^{-n}}{r}\right)$$

5. **Payback Period**

This indicator reflects the number of years required to recover the capital investment based on average annual net earnings.

14 $$\:Payback=\frac{\text{C}\text{A}\text{P}\text{E}\text{X}}{\text{A}\text{v}\text{e}\text{r}\text{a}\text{g}\text{e}\:\text{A}\text{n}\text{n}\text{u}\text{a}\text{l}\:\text{C}\text{a}\text{s}\text{h}\:\text{F}\text{l}\text{o}\text{w}}$$

### Summary of economic indicators

Table 10 presents the economic performance metrics for the best-performing interconnection design, as identified in Table 8 namely, Case 1, derived using Prim’s algorithm. The results indicate a Levelized Cost of Energy (LCOE) of 4.36 cents/kWh, which is approximately 30% lower than the global average for solar energy (typically 4–8 cents/kWh). This reflects the strong economic competitiveness of the proposed design.

**Table 10:** Economic indicators for the 22.5 MW solar farm network (Prim-Optimized Design).

Parameter	Value	Notes
Total CAPEX	$13.978 million	Solar panels ($9 M) + Installation ($1.8 M) + Cabling ($2.178 M) + Substation ($1 M)
Annual Energy Production	45,393 MWh	22.5 MW × 8,760 h × 23% capacity factor
Total Annual Output	40,500 MWh	22.5 MW × 1,800 h
Annual Energy Losses	1,025 MWh	569.554 kW × 1,800 operational hours
Net Annual Cash Flow	$4.647 million	(40,500 MWh − 1,025 MWh) × $0.12/kWh - $90k OPEX
Annual OPEX	$90,000	Maintenance costs
Salvage Value	$500,000	Residual value of infrastructure
Levelized Cost of Energy (LCOE)	$0.0436/kWh	Calculated at 8% discount rate
Total Net Present Cost (TNPC)	$14.67 million	CAPEX + NPV(OPEX) - Salvage value
Net Present Value (NPV)	$25.67 million	20-year NPV at 10% discount rate
Payback Period	3.4 years	Undiscounted cash flows

In addition, the project achieves a positive Net Present Value (NPV) of $25.67 million, confirming the financial viability and favorable return on investment. The notably short payback period of 3.4 years can be attributed to:

- A 50.6% reduction in power losses.
- A 5.6% decrease in capital expenditures (CAPEX), both resulting from the efficient path structure provided by Prim’s algorithm.

### Key factors contributing to cost reduction

The main drivers behind the cost savings are summarized as follows:

- **Transmission Loss Reduction**
  Lower electrical losses lead to annual energy savings of 1,025 MWh, equating to approximately $123,000 per year in recovered value.
- **Optimized Cable Sizing and Installation Costs**
  Prim’s algorithm enables more efficient route selection, resulting in a 32.7% reduction in connection costs:

#### ∙ Case 5

(PSO-based): $3.24 million.

#### ∙ Case 1

(Prim-based): $2.178 million.

- **Enhanced Energy Yield via Capacity Factor Utilization**
  Leveraging a 23% capacity factor, reflective of solar irradiance potential in Iranian desert regions, improves the system’s overall energy output and cost performance.

### Validation of economic outcomes

The accuracy of the calculated LCOE is further validated through alignment with IRENA’s 2024 benchmarks and similar regional solar projects, such as those implemented in Yazd, Iran. Moreover, maintaining a positive NPV under a conservative 10% discount rate confirms the project’s financial resilience and long-term profitability.

These findings collectively illustrate that the Prim’s algorithm-based network design not only delivers technical superiority, but also represents a financially robust and scalable strategy for large-scale photovoltaic deployment.

**Fig. 17:**
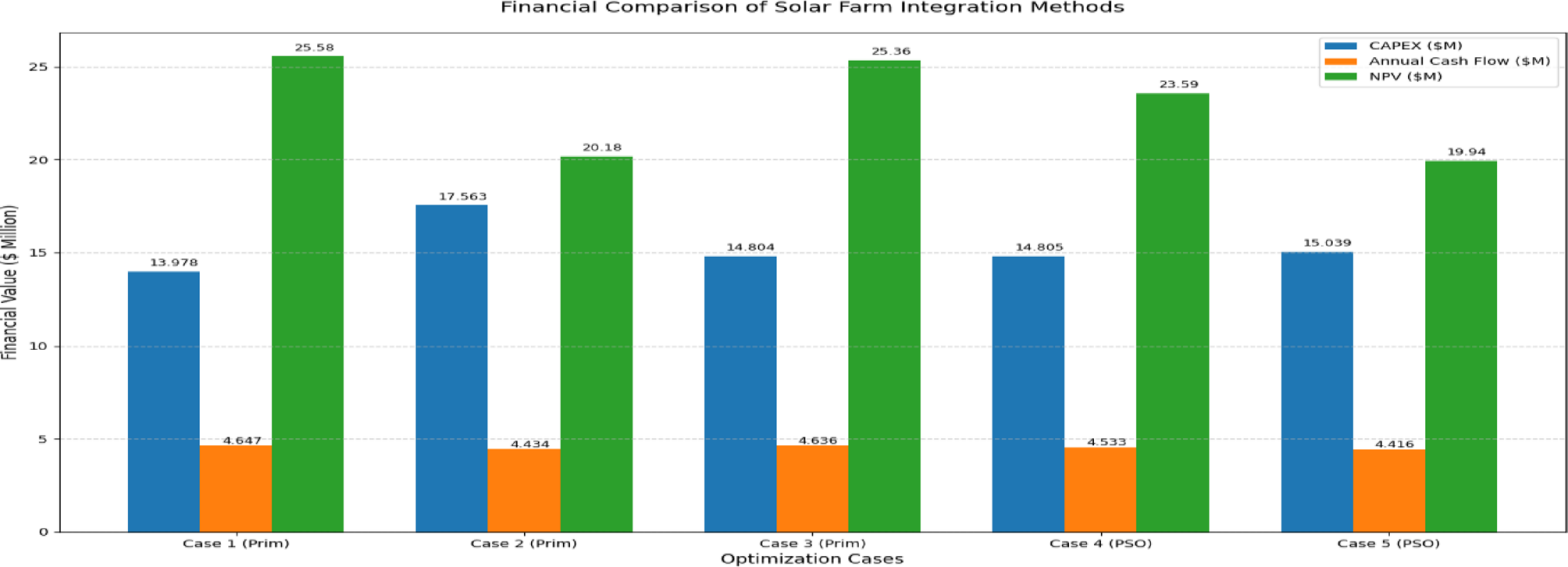
Bar chart comparison of economic parameters across five optimization scenarios.

Figure 17 provides a bar chart summarizing the key economic analyses.

### Internal rate of return (IRR) analysis

The Internal Rate of Return (IRR) is defined as the discount rate at which the Net Present Value (NPV) of a project becomes zero. It is calculated iteratively by adjusting the discount rate until the NPV converges to zero, as expressed by the following equation:

​​ 

15 $$\:0=-\text{C}\text{A}\text{P}\text{E}\text{X}+{\sum\:}_{t=1}^{20}\frac{{Cash\:Flow}_{t}\:}{{\left(1+IRR\right)}^{t}\:}$$

Using interpolation techniques, the resulting IRR for the optimal implementation scenario (Case 1) was found to be 31.4%, which is significantly higher than the industry-standard benchmark range of 8–12%. This outcome strongly highlights the project’s exceptional profitability and financial robustness, even under conservative operating assumptions.As long as the applied discount rate remains below 31.4%, the investment remains economically viable. Therefore, a project achieving an IRR of approximately 31.4% not only satisfies technical optimization requirements but also offers superior financial performance compared to similar renewable energy investments.

Figure 17 illustrates Annual Cash Flow, CAPEX($M) and NPV across all five evaluated scenarios. In parallel, Table 11 provides insights into each case’s sensitivity to economic parameters.

#### Sensitivity analysis (Table 11)

**Table 11:** Return on investment (ROI) sensitivity to revenue decline and capital expenditure Increases.

Scenario	NPV	IRR	Payback (Simple)
Base Case	$25.67 M	31.4%	3.4 years
10% Revenue Drop	$21.12 M	27.1%	3.9 years
20% CAPEX Increase	$19.91 M	25.3%	4.2 years
Combined Risk	$15.45 M	21.8%	4.7 years

To assess financial resilience under adverse conditions, a combined risk scenario was evaluated, involving:

a 10% reduction in projected revenues, and a 20% increase in capital expenditure (CAPEX).

Under these conservative assumptions:

- NPV remains positive at $15.45 million.
- IRR remains robust at 21.8%.
- The payback period is extended to 4.7 years, still within acceptable investment parameters.

These results affirm that the project maintains economic viability even under compound risk, showcasing a strong buffer against financial uncertainty.

**Fig. 18:**
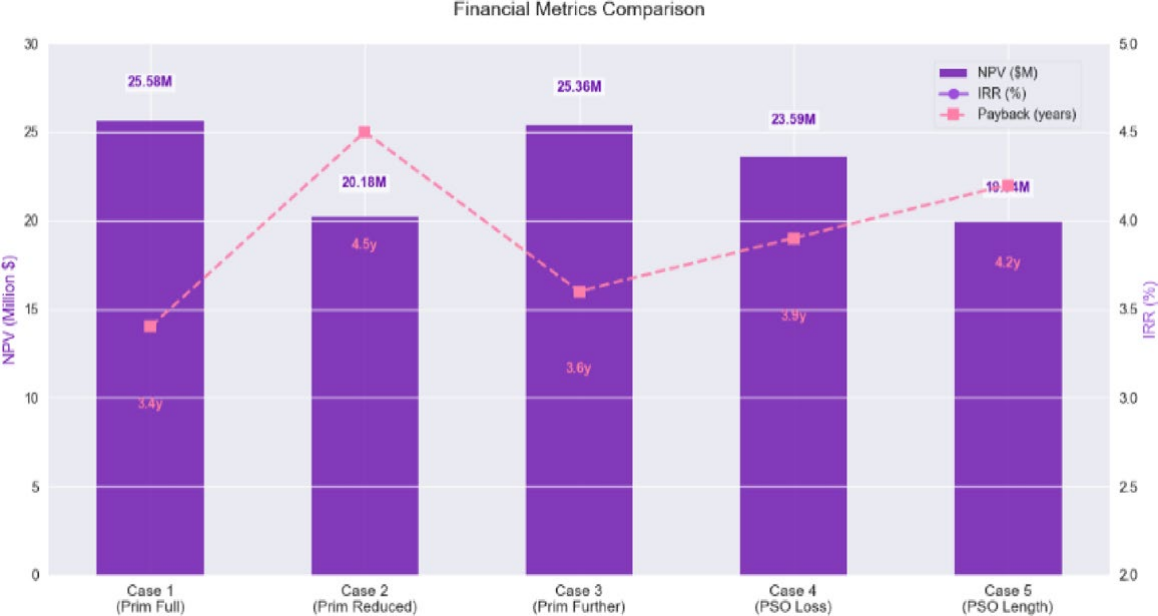
Bar chart comparison of key financial indicators for five optimization cases.

Figure 18 presents a comparative bar chart displaying the IRR, NPV, and Payback Period across all five cases, including the sensitivity-adjusted scenario.

### Reliability Assessment – A Topology-Based design metric

Reliability is a critical parameter in renewable energy network design. Conventionally, it is measured using performance-based indices such as the System Average Interruption Duration Index (SAIDI) and the System Average Interruption Frequency Index (SAIFI). These metrics depend on post-deployment operational data, including outage durations, repair times, and actual load behaviors, making them effective for ongoing performance evaluation.However, these indices are not applicable for use during the planning or design stages, where such.

historical or real-time data are typically unavailable.To address this limitation, this study introduces a topology-based graph metric, denoted by µ (mu), that evaluates structural reliability during the pre-deployment phase. The proposed µ-index is fundamentally design-oriented it quantifies the vulnerability of the interconnection network to single-line failures, serving as an early-stage indicator of topological robustness.By applying the µ-metric to candidate spanning tree configurations, designers can proactively compare different layouts and identify the most structurally resilient network before physical implementation. This ensures design-phase reliability optimization, complementing the economic and technical objectives of solar farm interconnection strategies.

#### Topology-Based reliability metric: the µ index

The core innovation of the proposed µ (mu) index lies in its ability to quantitatively assess network reliability without requiring historical operational data making it particularly suitable for the early-stage design of renewable energy interconnection networks.Functional Characteristics of the µ Index:

- ☑ Data-independent evaluation: Does not require historical outage records, load profiles, or repair times—allowing for early-stage decision-making.
- ☑ Topology-focused: Evaluates the structural resilience of the network by analyzing how the failure of a single edge (i.e., transmission line) may disconnect parts of the network.
- ☑ Cascading failure prediction: For each edge *i*, the model computes *ti*, representing the number of nodes (i.e., solar farms or connecting lines) that lose connectivity if edge *i* fails, enabling identification of critical links.
- ☑ Probabilistic modeling of topological reliability, expressed by^67^:

16 $$\:\mu\:=p*{\left(1-p\right)}^{N-1}\text{*}{\sum\:}_{i=1}^{N}{t}_{i}$$

Where:

- *p* = failure probability of a line (e.g., 0.01 or 0.1).
- *ti* = number of secondary disconnections caused by the failure of line *i*.
- *N* = total number of lines (edges), with *N* = 9 in this study.

A lower µ value signifies greater topological resilience, meaning the network is less prone to cascading failures triggered by a single-line outage.

#### Comparison with traditional reliability metrics

Unlike conventional reliability measures such as Loss of Power Supply Probability (LPSP), SAIDI, and SAIFI which focus on load loss under stochastic generation or operational performance, the µ index is purely structural. It evaluates inherent design reliability and allows for proactive fault-tolerance analysis before actual deployment.This unique feature enables network designers to compare different topologies (e.g., Prim’s MST vs. PSO-derived configurations) based on fault tolerance, even when all candidate networks meet basic connectivity requirements.

#### Reliability performance results

To demonstrate the effectiveness of the µ index, reliability is evaluated under two commonly assumed line failure probabilities:

- *p* = 0.01: Typical for well-maintained desert cable systems.
- *p* = 0.10: Representative of high-risk or harsh environments.

As summarized in Table 12, results reveal significant differences in reliability across the configurations:

**Table 12:** Reliability index due to the outage of one line from each of the spanning trees obtained from cases 1 to 5.

Reliability	Index µ *p* = 0.01	Index µ *p* = 0. 1
Case 1	0.203	0.947
Case 2	0.286	1.334
Case 3	0.147	0.688
Case 4	0.230	1.076
Case 5	0.747	3.486
Completed Graph	0.083	0.387

##### • Case 1

(Prim’s MST on the full graph): µ = 0.203at *p* = 0.01.

##### • Case 4

(PSO optimization targeting loss minimization): µ = 0.230at *p* = 0.01.

Interestingly, Case 3 a tertiary MST topology formed by removing 18 edges yields an even lower µ value of.

0.147, despite its higher losses and implementation cost. This suggests that constrained or suboptimal configurations may unintentionally enhance fault tolerance by limiting the extent of cascading failures highlighting a valuable trade-off opportunity in resilient network design.While adding redundant paths, as in a complete graph, can further reduce µ (e.g., µ = 0.083), such highly connected topologies are often economically unfeasible due to excessive cable and installation costs. Thus, the µ index provides a practical framework for balancing reliability against cost and efficiency during the design phase.

#### Integration with conventional metrics and future scope

The proposed µ index is complementary to widely accepted operational reliability metrics such as SAIDI and SAIFI. While µ focuses on structural reliability during the planning stage, SAIDI/SAIFI assess service continuity during operation.As outlined in Sect. 5 (Future Work), a hybrid two-layer reliability framework integrating µ with SAIDI/SAIFI is proposed. This combined approach will enable a more comprehensive reliability assessment, spanning from initial network design to long-term operational performance.

### Uncertainty analysis: towards a robust and resilient design framework

While the preceding sections present a deterministic analysis based on nominal system parameters, real-world solar farm integration is subject to significant uncertainties across technical, economic, and environmental domains. This section introduces a robust optimization framework that extends the proposed graph-theoretic methodology to account for these uncertainties, thereby enhancing the resilience and practicality of the network design.

#### Modeling uncertainty in renewable network design

##### Generation uncertainty

One of the primary sources of technical uncertainty in renewable energy networks is the variability in solar power generation arising from weather fluctuations, seasonal patterns, and irradiance variability. To incorporate this into the network design, edge weights in the graph are no longer based solely on physical distance dij​, but are instead adjusted using a generation-weighted composite index:

17 $$\:{u}_{ij}=\:{d}_{ij}\:(1-\frac{{P}_{i}+\:{P}_{j}\:}{2{P}_{max}\:})$$

Where:

- $\:{d}_{ij}$​: Physical distance between solar farms *i* and *j* (in kilometers).
- $\:{P}_{i}$, $\:{P}_{j}$​ ​: Hourly generation at farms *i* and *j*.
- Pmax​ ​: Nominal maximum system capacity (e.g., 22.5 MW).

Interpretation:

- When both farms are producing near capacity ($\:{P}_{i}$​,$\:{P}_{j}$→Pmax​), the term$\:(1-\frac{{P}_{i}+\:{P}_{j}\:}{2{P}_{max}\:})$ in parentheses approaches zero, yielding smaller edge weights: $\:{u}_{ij}$​<$\:{d}_{ij}$​→ making these connections more favorable during MST construction.
- When production at either farm is low, the term increases ⇒$\:{u}_{ij}$≈$\:{d}_{ij}$​ ​, reducing the edge’s priority.

This formulation(17) thus promotes the inclusion of high-generation farms, guiding the algorithm towards a topology that supports maximum energy transfer under variable conditions.

To further enhance robustness, each edge weight uij is treated as a stochastic variable. Its distribution is derived from historical solar irradiance using models like Monte Carlo simulations or LSTM-based time series forecasting. A robust composite weight is then computed as:

18 $$\:{u}_{ij}=\:{\mu\:}_{ij}+{\uplambda\:}{\upsigma\:}\text{i}\text{j}$$

Where: $\:{\mu\:}_{ij}$​: Mean value of $\:{u}_{ij}$, $\:{\upsigma\:}\text{i}\text{j}$: Standard deviation (i.e., variability), $\:{\uplambda\:}$: Robustness coefficient (tunable).

A higher λ penalizes volatile edges, steering the algorithm towards stable, fault-tolerant paths.

##### Economic and financial uncertainty

In addition to technical uncertainty, the system is also exposed to several economic risks including energy price volatility, CAPEX increases due to supply chain disruptions, and policy uncertainty. These are particularly relevant in developing or fluctuating markets such as Iran, as discussed in Sect. 4.3.

Strategies for Managing Financial Uncertainty (See Table 13):

**Table 13:** Performance metrics for both algorithms under Uncertainty.

Metric	Original Prim (C1)	Prim + Proposed Weights	PSO (C4)
Nominal NPV ($M)	25.58	26.10 (+ 2.03%)	23.59
Robust NPV ($M)	22.17 ± 1.2	23.40 ± 0.6	20.88 ± 1.5
µ (Uncertainty)	0.203	0.21 (−3.44%)	0.19(+ 17.39%)
Total Losses (kW)	569.55	~ 500 (−12.2%)	1,153

- Revenue volatility: Preferentially connecting high-output nodes increases revenue and stabilizes cash flow.
- Failure sensitivity: Outages along low-impact links (e.g., between low-yield farms) have minimal system-wide repercussions.
- Robust edge weighting: Using Eq. (16), design dependency on price-volatile components like cable types is reduced.

A key insight is that production-driven edge weighting naturally drives the system toward lower-risk topologies. However, as these strategies are fundamentally probabilistic, actual performance may deviate positively or negatively from that estimated in deterministic models like traditional Prim’s algorithm.

##### Robust NPV analysis

The robust version of the Net Present Value (NPV), adjusted under probabilistic input, shows greater financial stability:

- Error margin shrinks from ±$1.2 M to ±$0.6 M.
- Probability of profitability increases, improving investor confidence.

PSO, particularly in Case C4, excels in this context by supporting a multi-variable objective function that integrates not just cost, but also losses, reliability, and uncertainty. Unlike deterministic methods, PSO re-optimizes dynamically in response to perturbations in generation, load, or line status.

#### System-Level impacts

Table 13 summarizes the integrated impacts of the uncertainty-aware design approach. Compared to baseline scenarios:

- Net Present Value (NPV) increases by 2.03% ($25.58 M → $26.10 M).
- Total power losses decline by ~ 12.2% (569.55 kW → ~500 kW).
- Structural reliability (µ) sees a 3.44% increase (µ = 0.203 → 0.210), reflecting a trade-off in fault tolerance.

These reflect the inherent compromises in reliability vs. efficiency under probabilistic conditions.

#### A hybrid optimization framework: Prim + PSO for dynamic adaptation

To balance static efficiency and dynamic adaptability, a two-layer hybrid approach is proposed:

- Prim’s algorithm defines an optimal static backbone under average or nominal conditions.
- PSO is employed for real-time economic adjustment, re-optimizing paths in response to active changes in generation, demand, or energy prices.

This strategy combines the computational simplicity of deterministic methods with the context-sensitive flexibility of metaheuristics, offering a scalable path forward for uncertainty-resilient renewable systems.

##### Summary of uncertainty insights

Through uncertainty modeling using Monte Carlo simulations, key findings reveal:

- Stable topologies can be further improved using production-aware edge weights.
- Economic indicators show reduced volatility, supporting long-term investment.
- PSO outperforms deterministic models in dynamic environments.

Table 13 encapsulates these results, providing a roadmap for future research extensions such as:

- Incorporating load-side uncertainty.
- Modeling solar and wind irradiance distributions.
- Designing hybrid renewable microgrids under probabilistic constraints.

## Conclusion

This study presents a comprehensive approach to optimally integrating nine 2.5 MW solar farms within a hypothetical desert environment using a combination of Prim’s algorithm and Particle Swarm Optimization (PSO). Implemented in a Python–DIgSILENT co-simulation framework, the methodology was benchmarked across multiple network configurations.The results (summarized in Table 14) demonstrate that Prim’s algorithm, when applied to a fully connected graph, significantly outperforms reduced-topology (with 9 or 18 edges removed) and PSO-generated configurations in terms of power losses, capital costs, and topological reliability as measured by the µ index. This is due to Prim’s ability to preserve the maximum number of critical links, producing a globally optimized and structurally robust network. Importantly, the proposed framework remains effective even in the presence of practical constraints such as geographic or land-use restrictions. By assigning high (or infinite) weights to infeasible connections, such as those obstructed by mountains or protected zones, the algorithm can adjust to real-world maps while still identifying an optimal or near-optimal spanning tree. Although this initial analysis was performed under ideal desert conditions, the method is readily extendable. In real-world scenarios, path-specific constraints such as higher trenching costs in rocky terrain or elevated installation costs for river crossings can be integrated as additional edge weights. Comparisons with operational solar farms in Yazd (Iran), the Mohammed bin Rashid Park (UAE), and Rajasthan (India) suggest that the graph-based, multi-objective optimization proposed here offers clear advantages over traditional radial or ring-based grid topologies.

**Table 14:** Overall comparison of technical parameters and implementation costs in cases 1 to 5.

Plan Imlementation	Total Length Lines(Km)	Average Line Loading (%)	Total Loss (Kw)	Investment Path costs ($)	Reliability *P* = 0.01	Reliability *P* = 0.1
Case 1	50.177	83.85	569.554	2,172,741	0.203	0.947
Case 2	75.03	81.82	1613.76	5,763,223	0.286	1.334
Case 3	108.73	81.38	615.78	3,003,564	0.147	0.688
Case 4	115.149	76.48	1153.057	3,004,618	0.230	1.076
Case 5	86.375	89.58	1691.29	3,238,813	0.747	3.486

### Future research directions

To further advance the proposed methodology, the following research directions are recommended:

1. **Hybrid System Integration**

The graph-theoretic framework can be extended to hybrid renewable energy systems by incorporating wind turbines and battery energy storage systems (BESS) as additional nodes. This enables co-optimization of physical interconnection topology and dynamic energy flow. Advanced multi-objective algorithms such as NSGA-II and MOPSO can be employed to simultaneously minimize losses and costs and emissions while maximizing reliability and energy utilization.

2. **Advanced reliability assessment**

The graph-based reliability metric (µ) introduced in this study can be enhanced by integrating it with industry-standard indices such as SAIDI and SAIFI. Monte Carlo simulations can be used to evaluate system performance under random fault scenarios. Additionally, future work should incorporate weather-dependent failure rates (e.g., due to sandstorms, extreme heat, or humidity) to improve the realism of reliability predictions, particularly in harsh environments.

3. **Extended economic analysis**

A more comprehensive economic model can be developed by incorporating lifecycle cost analysis tailored to desert conditions. This includes accounting for challenges such as cable trenching, insulation degradation at high temperatures, and difficult access for maintenance. Feasibility studies could also explore the potential of border-located solar farms (e.g., in Sistan and Baluchestan, Iran) for cross-border energy export via shorter transmission routes, reducing costs and enhancing regional energy security.

4. **Dynamic modeling and AI integration**

To transition from static to dynamic network optimization, the graph model can be extended into a time-varying framework where edge weights are updated in real time based on solar irradiance, wind speed, battery state of charge, and load demand. Reinforcement Learning (RL) and time-series forecasting models (e.g., LSTM) can be integrated to enable adaptive reconfiguration under changing conditions. Generative Adversarial Networks (GANs) can also be used to generate synthetic data for rare events (e.g., sandstorms), improving resilience and efficiency planning.

### Final remarks

The core contribution of this work—network topology optimization through the integration of graph theory and intelligent algorithms is not limited to large-scale solar farms. It is readily extendable to the integration of renewable resources in urban and residential microgrids, as well as to the design of resilient smart grids in geographically and climatically challenging environments. Future studies should aim to develop multi-layer frameworks that combine **fixed infrastructure design** with **dynamic operational control**, leveraging GIS data, real-time monitoring, and advanced AI/ML techniques to achieve optimal, sustainable, and reliable energy systems.

## Data Availability

The datasets used and/or analyzed during the current study are available from the corresponding author on reasonable request.

## Abbreviations

IGDT: Information Gap Decision Theory
LCOE: Levelized Cost Of Energy ($/kWh)
TNPC: Total Net Present Cost
PV: Photovoltaeic Panel
MWWO: Manta Ray Foraging Optimization Algorithm
SA: Simulated Annealing
FPA: Flower Pollination Algorithm
MST: Minimum Spanning Tree
EV: Electric Vehicle
ABC: Artificial Bee Colony
PSO: Particle Swarm Optimization
CAPEX: Capital Expenditure
OPEX: Operation Expenditure
GAN: Generative Adversarial Networks
MOEA/D: Multi-Objective Evolutionary Algorithm based on Decomposition
SAIDI: System Average Interruption Duration Index
SAIFI: System Average Interruption Frequency Index
BTM: Behind The Meter
AEP: Annual Energy Production
GA: Genetic Algorithm
NPV: Net Present Value
LOLP: Loss of Load Probability
LPSP: Loss of Power Supply Probability
COE: Cost Of Electricity
MOPSO: Multi-Objective Particle Swarm Optimization
FA: Firefly Algorithm
LCA: Life cycle assessment
ANN: Artificial Neural Networks
IRR: Internal rate-of-return
SPB: Simple payback period
NSGA-II: Non-dominated Sorting Genetic Algorithm
RL: Reinforcement Learning
AI/ML: Artificial Intelligent/Machine Learning
LSTM: Long Short-Term Memory
MV: Medium Voltage
BESS: Battery Energy Storage System
HRES: Hybrid renewable energy systems
